# Dietary Fish Hydrolysate Improves Memory Performance Through Microglial Signature Remodeling During Aging

**DOI:** 10.3389/fnut.2021.750292

**Published:** 2021-11-23

**Authors:** Mathilde Chataigner, Céline Lucas, Mathieu Di Miceli, Véronique Pallet, Sophie Laye, Alexis Mehaignerie, Elodie Bouvret, Anne-Laure Dinel, Corinne Joffre

**Affiliations:** ^1^Université de Bordeaux, INRAE, Bordeaux INP, NutriNeuro, Bordeaux, France; ^2^Abyss Ingredients, Caudan, France; ^3^NutriBrain Research and Technology Transfer, NutriNeuro, Bordeaux, France; ^4^Worcester Biomedical Research Group, School of Science and the Environment, University of Worcester, Worcester, United Kingdom

**Keywords:** n-3 long chain PUFA, low molecular weight peptides, microglia, memory, hydrolysate, cognitive decline, aging

## Abstract

Brain aging is characterized by a chronic low-grade inflammation, which significantly impairs cognitive function. Microglial cells, the immunocompetent cells of the brain, present a different phenotype, switching from a homeostatic signature (M0) to a more reactive phenotype called “MGnD” (microglial neurodegenerative phenotype), leading to a high production of pro-inflammatory cytokines. Furthermore, microglial cells can be activated by age-induced gut dysbiosis through the vagus nerve or the modulation of the peripheral immune system. Nutrients, in particular n-3 long chain polyunsaturated fatty acids (LC-PUFAs) and low molecular weight peptides, display powerful immunomodulatory properties, and can thus prevent age-related cognitive decline. The objective of this study was to investigate the effects of n-3 LC-PUFAs and low molecular weight peptides contained in a marine by-product-derived hydrolysate on microglial phenotypes and intestinal permeability and their consequences on cognition in mice. We demonstrated that the hydrolysate supplementation for 8 weeks prevented short- and long-term memory decline during aging. These observations were linked to the modulation of microglial signature. Indeed, the hydrolysate supplementation promoted homeostatic microglial phenotype by increasing TGF-β1 expression and stimulated phagocytosis by increasing Clec7a expression. Moreover, the hydrolysate supplementation promoted anti-inflammatory intestinal pathway and tended to prevent intestinal permeability alteration occurring during aging. Therefore, the fish hydrolysate appears as an interesting candidate to prevent cognitive decline during aging.

## Introduction

Brain aging has been associated with a chronic low-grade inflammation, in humans (1–3) and rodents (4–6). Neuroinflammation is finely orchestrated by microglial cells, the immunocompetent cells of the central nervous system (CNS). In the healthy brain, microglial cells exhibit a unique molecular homeostatic signature (M0) but with aging, these cells can display a novel non-homeostatic signature called “MGnD” (microglial neurodegenerative phenotype) and become sensitized to inflammation and highly reactive, leading to an imbalance between pro- and anti-inflammatory cytokine production (7, 8). During aging, microglial cells express pro-inflammatory markers such as galectin 3 (Lgals3), the AXL receptor tyrosine kinase (Axl), c-type lectin domain family 7-member A (Clec7a), the major histocompatibility complex class II (MHCII) and the integrin subunit alpha X (Itgax also known as CD11c) (9–11). Moreover, transforming growth factor β (TGF-β), an important molecule in the maintaining of the M0 phenotype, is decreased in microglial cells of aged mice, contributing to the shift toward MGnD signature (7, 10). Microglia can also be activated by aged-induced gut dysbiosis. Indeed, aging has been linked to a decrease of gut microbiota diversity and an increase of intestinal permeability and inflammation, contributing to microglia activation *via* the vagus nerve or by direct modulation of the peripheral immune system (12–15).

This microglial dysfunction can lead to aged-related cognitive decline which is characterized by non-pathological, but significant alterations of memory in both humans and animals (16, 17). This cognitive decline can lead to alteration of well-being and quality of life (18, 19). Indeed, in humans, a mild stimulation of the host defense is associated with increased cytokine release and negative effects on emotional and memory functions (20). In rodents, interleukin (IL)-1β injection induces decreased memory performance as measured in an 8-arm radial maze or in the Morris water maze (21, 22). Moreover, spatial memory is altered in transgenic mice overexpressing tumor necrosis factor α (TNF-α) in the brain, while it is enhanced in TNF-α deficient mice (23, 24). Thus, targeting inflammation during aging constitutes a good strategy to delay or limit the development of age-related cognitive deficits (25).

Nutrition is an innovative strategy to prevent age-related cognitive impairments. Among all nutrients, n-3 long chain polyunsaturated fatty acids (LC-PUFAs) and low molecular weight peptides derived from proteins are good candidates for their immunomodulatory properties. n-3 LC-PUFAs, including docosahexaenoic acid (DHA) and eicosapentaenoic acid (EPA), display powerful anti-inflammatory and pro-resolutive properties. Indeed, they regulate the release of pro-inflammatory mediators, as evidenced in clinical and pre-clinical *in vivo* studies, as well as in *in vitro* studies (26–28). In humans suffering from diseases associated with chronic low-grade inflammation, supplementations with EPA and/or DHA reduce circulating pro-inflammatory cytokines expression and increase the production of specialized pro-resolving mediators (SPM) (29–31). Supplementation with n-3 LC-PUFAs in adult rodents prevents the increase of the pro-inflammatory cytokine expression IL-1β, IL-6 and TNF-α induced by lipopolysaccharide (LPS) or IL-1β and increases hippocampal production of anti-inflammatory cytokines, such as IL-10 and IL-4 (32–38). Furthermore, numerous observational and interventional studies highlighted the positive association between the consumption of dietary n-3 LC-PUFAs and cognitive performance in the elderly (39–43). Similarly, beneficial effect of n-3 LC-PUFAs supplementations on cognition have also been shown in aged rodents (44–47). Aged mice supplemented with DHA and/or EPA are protected against neuroinflammation and cognitive impairment (46). *In vitro*, anti-inflammatory effects of n-3 LC-PUFAs have been demonstrated in microglial cells with the reduction of LPS-induced expression of pro-inflammatory cytokines as well as the polarization of microglial cells into an anti-inflammatory phenotype (48–54). Low molecular weight peptides (<1,000 Da) contained in protein hydrolysates are also nutrients of interest for their central and peripheral anti-inflammatory properties, demonstrated *in vivo* and *in vitro* (55–59). In a mouse model of Alzheimer's disease, peptides from milk reduced the expression of inflammatory factors such as TNF-α, monocyte chemoattractant protein-1 (MCP-1/CCL2) inducible nitric oxide synthase (iNOS) in the brain (60). *In vitro*, in human primary monocytes and murine macrophages, salmon- and lupine-derived peptides inhibited the production of nitric oxide (NO), prostaglandin (PG) E2 and pro-inflammatory cytokines, including TNF-α, IL-6, and IL-1β (61, 62). At the periphery, peptides from soy and milk reduced peripheral expression of pro-inflammatory factors such as TNF-α, IL-6, IL-1β, interferon-γ, or IL-17 in mice colon and abdominal aorta (56, 63). Furthermore, at the intestine level, an intake of marine n-3 PUFAs or bioactive peptides (from soy or oyster hydrolysate, for example) has been shown to decrease intestinal inflammation induced by inflammatory bowel diseases in humans and mice (64, 65). n-3 LC-PUFAs have been shown to influence the gut microbiota and improve intestinal immunity (66, 67). In rodents, supplementation with n-3 LC-PUFAs increases the number and abundance of beneficial bacteria, such as *Bifidobacterium* (68, 69). EPA and DHA have also been shown to prevent intestinal permeability changes induced *in vitro* and *in vivo* (70). Moreover, low molecular weight collagen peptides have been shown to protect the intestinal barrier function *in vitro via* the regulation of tight junction proteins zonula occludens 1 (ZO-1) and occludin (Ocln) expression and distribution and the myosin light chain kinase (MLCK) pathway (71, 72).

In this study we investigated the effects of n-3 LC-PUFAs and low molecular weight peptides contained in a marine by-product-derived hydrolysate on microglial signature, intestinal permeability, and cognition in mice.

## Materials and Methods

### Animals

Fifteen-month old male C57Bl6/J mice (Janvier Labs, Le Genest-Saint-Isle, France) were housed under normal 12 h-12 h light/dark cycle on cellulose litter in a controlled environment (21–23°C, 40% of humidity), with *ad libitum* access to food and water. Animal husbandry and experimental procedures were done in accordance with the EU Directive 2010/63/EU for animal experiments and were approved by the local ethical committee (CE050 from Bordeaux) for the care and use of animals (approval ID APAFIS#14144-2018041213072383).

### Diet

**Figure 1:**
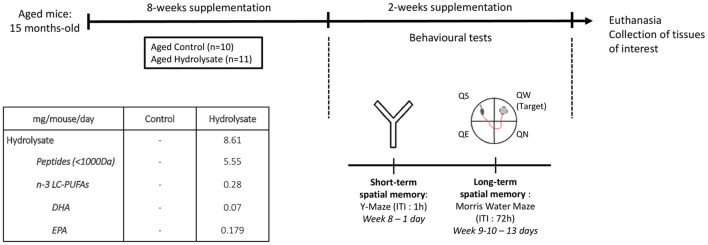
Experimental design. Aged mice (15 months) were fed with the control diet or with the hydrolysate-enriched diet for 8 weeks. Behavioral tests were performed during the next 2 weeks. Total supplementation duration was 10 weeks. DHA, docosahexaenoic acid; EPA, eicosapentaenoic acid; ITI, inter-trial interval; LC-PUFAs, long chain polyunsaturated fatty acids; QE, quadrant east, QN, quadrant north; QS, quadrant south; QW, quadrant west.

Mice were randomly assigned to different groups: one group (*n* = 10) fed a control diet (INRAE Jouy-en-Josas, France) and one group (*n* = 11) fed the hydrolysate-enriched diet (INRAE Jouy-en-Josas, France) containing 0.29% of the fish hydrolysate for 8 weeks (Table 1; Figure 1). The fish hydrolysate was provided by the BrainBooster Consortium. It was obtained from marine by-products and contained mostly low molecular weight peptides (<1,000 Da) and n-3 LC-PUFAs such as DHA and EPA. The specific composition of the fish hydrolysate is detailed in patent number B251427FR. The fish hydrolysate dose was determined as previously shown (73). The dose of low molecular weight peptides was 5.55 mg/mouse/day, and the dose of n-3 LC-PUFAs was 280 μg/mouse/day (of which 70 μg/mouse/day of DHA and 179 μg/mouse/day of EPA).

**Table 1:** Composition of the control and the hydrolysate-enriched diets.

**Components**	**Percent (%)**	
	**Control diet**	**Hydrolysate-enriched diet**
Hydrochloric casein	18	18
Corn starch	45.9	45.7
Sucrose	24	24
Cellulose	2	2
Peanut oil	5	5
Mineral mix	4	4
Vitamin mix	1	1
+ DL methionine	0.1	0.1
+ Vitamin A 5 UI/g	5 UI/g	5 UI/g
Hydrolysate	0	0.29

### EchoMRI

Fat mass and lean mass were quantified at the beginning and at the end of the supplementation by magnetic resonance using minispec LF90 II (Bruker, Wissembourg, 67166).

### Behavioral Tests

#### Y-Maze

Eight weeks after the beginning of the supplementation, short term spatial recognition memory was evaluated with the Y-maze test as described previously (74). The apparatus is a Y-shaped maze with 3 arms (35 cm long and 10 cm deep), illuminated at 15 lx. Extra-maze visual cues are placed on the walls, allowing mice to navigate in space. In the first trial, one arm of the Y-maze was closed, and mice were allowed to visit the two other arms for 5 min. Short term spatial memory was evaluated after a 1 h inter-trial interval (ITI), where mice were placed again in the start arm for the second trial and allowed to explore freely all three arms for 5 min. Start and closed arms were randomly assigned for each mouse. The animals were video-tracked (SMART system; Bioseb, Vitrolles, France) to analyze the distance traveled in the different arms.

#### Morris Water Maze

Spatial learning and memory were assessed in the Morris water maze as previously detailed (73, 75). Briefly, two familiarization days were designed. Mice had to find a submerged platform in a small pool (60 cm diameter) to familiarize with water and swimming (3 consecutive trials a day; 60 s cut-off). Then, visuomotor deficits were evaluated during a day of cued learning in the Morris water maze where mice had to find a submerged platform pointed out with a cue (6 trials a day; 90 s cut-off). During spatial learning, mice were trained during four consecutive days to learn the location of the submerged platform by using distal extra-maze cues (6 trials a day; 90 s cut-off). For each trial, the distance traveled to reach the platform was recorded by Imetronic videotracking system (Pessac, France). Spatial memory was assessed 72 h after the last day of training, during the probe test for 60 s in the maze without the platform. The distance traveled in the four quadrants was recorded using the SMART system (Bioseb, Vitrolles, France). The quadrant containing the platform is referred to as “target quadrant.”

### Tissue Preparation

Mice were euthanized by injection of a cocktail of ketamine/xylamin the day following the probe test. After transcardiac perfusion with phosphate buffered saline (PBS), brain structures and peripheral organs of interest were collected and frozen at −80°C until further analysis. For the immunohistochemistry analysis, hemispheres were post-fixed in 4% paraformaldehyde (PFA) overnight at 4°C, cryoprotected in 30% sucrose during 48 h at 4°C, rapidly frozen with isopentane and stored at −80°C.

### Biochemical Measurements

#### Quantitative Real-Time PCR

The expression of the different genes of interest was evaluated by real time quantitative PCR as previously described previously (73). These analyses were performed on central (hippocampus) and peripheral structures (ileum and colon). Briefly, total RNAs were extracted from hippocampus, ileum and colon by TRIzol (Invitrogen, Life Technologies, Saint Aubin, France). Quantity and purity of RNA for each sample were measured by spectrophotometry (Nanodrop, Life technologies, Saint Aubin, France). Reverse transcription was performed on one or two micrograms of RNA by Superscript IV (Invitrogen, Life Technologies, Saint Aubin, France). TaqMan^®^ specific primers were used to amplify genes of interest as previously described (73). We focused on IL-6 (Mm00446190_m1), IL-1β (Mm00434228_m1), TNF-α (Mm00443258_m1), TGF-β1 (Mm01178820_m1), transforming growth factor β receptor 2 (TGF-βr2; Mm03024091_m1), αM integrin (Itgam; Mm00434455_m1); transmembrane protein 119 (Tmem119; Mm00525305_m1), P2Y purinoceptor 12 (P2y12; Mm00446026_m1), colony-stimulating factor 1 receptor (CSF1r; Mm01266652_m1), MHCII (Mm00439216_m1), triggering receptor expressed on myeloid cells 2 (Trem2; Mm04209424_g1), Apolipoprotein E (ApoE; Mm01307193_g1), Lgals3 (Mm00802901_m1), Axl (Mm00437221_m1), Clec7a (Mm01183349_m1), Itgax (Mm00498708_g1), IL-10 (Mm01288386_m1), Ocln (Mm00500912_m1), ZO-1 (Mm00493699_m1), claudin 5 (Cldn5; Mm00727012_s1), and MLCK (Mm00653039_m1). The housekeeping gene was β-2-microglobulin (B2m; Mm00437762_m1). Fluorescence was determined on a LightCycler^®^ 480 instrument II (Roche, La Rochelle, France). Data were analyzed using the comparative threshold cycle (Ct) method and results were expressed as relative fold change (73, 76, 77) to control target mRNA expression.

#### Immunohistochemistry

Free-floating coronal sections of 40 μm through the hippocampus were collected on a cryostat (Leica Biosystems, Nanterre, France). After being washed for 10 min with PBS-Tween 0.01%, sections were blocked in a buffer containing 5% of donkey serum, 5% of bovine serum albumin (BSA), 0.3% Triton in PBS 1X for 1 h at room temperature (RT). Sections were then immunolabelled with a rabbit polyclonal antibody against Iba1 (1:1,000; Wako #019-19741, Plaisir, France) and a rat polyclonal antibody Clec7a (1:50, Invitrogen, Life Technologies # MABG-MDECT, Saint Aubin, France) in a staining buffer containing 5% of BSA, 0.1% of triton in PBS 1X over night at 4°C. After being washed in PBS-Tween 0.01%, slices were incubated with donkey anti-rabbit 488 (1:2,000; Invitrogen, Life Technologies #A-21206, Saint Aubin, France) and donkey anti-rat 594 (1:100, Invitrogen, Life Technologies #A-21209, Saint Aubin, France) secondary antibodies in a buffer containing 5% of BSA in PBS 1X for 2 h at RT. All sections were processed in parallel. Staining was visualized using DAPI (Santa Cruz Biotechnology, Heidelberg, Germany). Images were obtained with a 20 × microscope objective and the software NIS-Elements AR3-2 (Nikon Eclipse 400, Nikon Corporation, Champigny-sur-Marne, France). The number of Iba1- and Clec7a-positive cells in the hippocampus was counted using Image J software (Image J, open source).

#### Western Blot

Proteins were extracted from the TRIzol fraction previously recovered from the RNA extraction step using the extraction protocol of Simões et al. (78). Protein concentration was determined by bicinchoninic acid protein assay (Interchim, Montlucon, France) according to the protocol. For analysis, proteins were resolved on 10% sodium dodecyl sulfate-polyacrylamide gel and transferred to nitrocellulose membranes. Membranes were incubated with different primary antibodies: a rabbit polyclonal anti-ZO-1 (1:500, #61-7300, Invitrogen, Life Technologies, Saint Aubin, France), a rabbit polyclonal anti-ocln (1:250, #40-4700, Invitrogen, Life Technologies, Saint Aubin, France) and a rabbit polyclonal anti-GAPDH as housekeeping protein (1:10,000; #51745, Cell Signaling, Leiden, Netherlands). These primary antibodies were detected with appropriated donkey horseradish peroxidase-conjugated secondary antibodies (1:5,000, #711-035-152, Jackson Immunoresearch, Westgrove, PA, USA). The membranes were incubated with a peroxidase revealing solution (SuperSignal West Dura, ThermoFisher, Waltham, MA, USA) and were revealed using ChemiDoc MP (Biorad, Hercules, CA, USA). Proteins of interest were normalized to GAPDH and results are expressed as relative expression.

### Data Analysis

Hierarchical cluster analysis was performed using R free software (79), version 4.0.3. Forty variables were used (Table 2). Then, unsupervised hierarchical analysis was performed with *hclust* function (80) using Ward's linkage method (81). The resulting cluster dendrogram was then generated with the *plot* function. Correlation matrices were calculated and drawn in R with *heatmap.plus, gplots, psy, RcolorBrewer, corrplot, ggplot2, Hmisc* and *ggcorrplot* packages (cran.r-project.org). All aforementioned packages can be found on the CRAN repository (https://cran.r-project.org/).

**Table 2:** Variables used for hierarchical cluster analysis.

**Family**		**Process**	**Variable**	**Full name**
Central nervous system		Inflammation	IL6	Interleukin 6
			IL1b	Interleukin 1β
			TNFa	Tumor necrosis factor α
M0		Microglial phenotype	TGFb1	Transforming growth factor β1
			TGFbr2	Transforming growth factor β receptor 2
			Itgam	αM integrin
			Tmem119	Transmembrane protein 119
			P2Y12	P2Y purinoceptor 12
			CSF1r	Colony-stimulating factor 1 receptor
MGnD		Microglial phenotype	MHCII	Major histocompatibility complex class II
			Trem2	Triggering receptor expressed on myeloid cells 2
			ApoE	Apolipoprotein E
			Lgals3	Galectin 3
			Axl	Tyrosine-protein kinase receptor UFO
			Clec7a	C-type lectin domain containing 7A
			Itgax	αX integrin
			Clec7a^+^ Iba1^+^/Iba1^+^	C-type lectin domain containing 7A Ionized calcium binding adapter molecule1
Intestinal tract	Colon	Inflammation	IL6	Interleukin 6
			IL1b	Interleukin 1β
			TNFa	Tumor necrosis factor α
			IL10	Interleulin 10
		Permeability	Protein Ocln	Protein occludin
			Protein ZO-1	Protein ZO-1
			Ocln	Occludin
			ZO-1	Zonula occludens-1
			Cldn5	Claudin 5
			MLCK	Myosin light-chain kinase
	Ileum	Inflammation	IL6	Interleukin 6
			IL1b	Interleukin 1β
			TNFa	Tumor necrosis factor α
			IL10	Interleukin 10
		Permeability	Protein Ocln	Protein occludin
			Protein ZO-1	Protein ZO-1
			Ocln	Occludin
			ZO-1	Zonula occludens-1
			Cldn5	Claudin 5
			MLCK	Myosin light-chain kinase
Behavior		Cognition	Distance target	Distance in the target quadrant of the MWM
			Y.Maze. New arm	Distance in the new arm of the Y-Maze
			Y.Maze. Familiar arm	Distance in the familiar arm of the Y-Maze

*M0, Microglial homeostatic signature; MGnD, Microglial neurodegenerative-associated disease phenotype*.

Statistical analyses were conducted with GraphPad Prism 7 (GraphPadSotfware, San Diego, USA). Graphs are represented as mean ± standard error of the mean (SEM). A 2-way ANOVA with repeated measures was used to analyze body weight (factors: diet and time). The Y-Maze was analyzed using a 2-way ANOVA followed by a Tukey *post-hoc* test. Concerning the Morris water maze:

- Cued learning was analyzed using an unpaired *t*-test.
- Spatial learning was analyzed using a 2-way ANOVA with repeated measures (factors: diet and days of learning).
- Probe test comparisons were performed for each group against chance level (25%) using a one sample *t*-test and a 1-way ANOVA (factor: quadrants) followed by a Dunnett's multiple comparisons *post-hoc* test. A 2-way ANOVA has also been performed (factors: quadrant and diet).

The other analyses were performed using unpaired *t*-tests (when variances were not different) or Welch-corrected *t*-tests (when variances were different) between groups. For the ANOVA analyses, the method of Geisser-Greenhouse was used to correct the violation of the assumption of sphericity (82). Alpha has been set at 0.05 and all the *post-hoc* tests used in the present study (Tukey's and Dunnett's) are comparing multiple variables and are also correcting for family wise error rate.

## Results

### Individual Heterogeneity in the Experimental Groups

**Figure 2:**
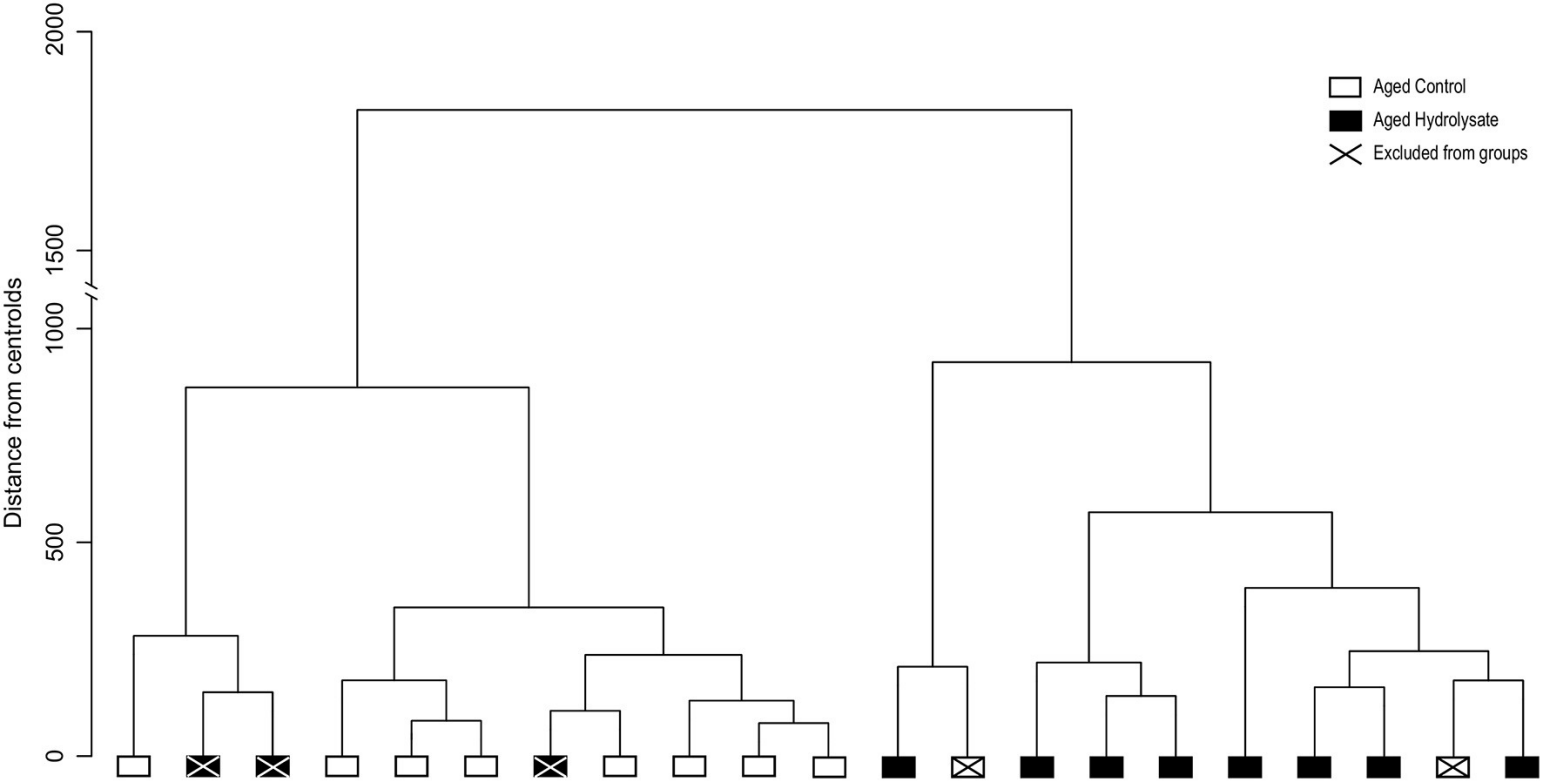
Unsupervised hierarchical cluster dendrogram of individuals using Ward's linkage method. Dendrogram of the two clusters corresponding to control fed mice (white) and mice fed the hydrolysate-enriched diet (black). Mice removed from analyses are represented with a cross. Such analyses are based on cognitive behavior, central and intestinal inflammatory cytokines, microglial and intestinal permeability markers (Table 2).

Unsupervised hierarchical clustering analysis was performed using input from all the behavioral and biochemical parameters previously described (Table 2). Output clustered mice into two different clusters. The majority of mice that were given hydrolysate-enriched or control diets were segregated in two separate clusters (Figure 2). However, out of 11 mice fed with the hydrolysate enriched-diet, 3 mice did not behave as the majority of the group. Similarly, 2 out the 10 mice fed with the control diet did not behave as the majority of the group. Subsequently, these mice were considered as not homogenous within their respective groups and were therefore considered as outliers, and excluded from further analyses (Figure 2).

### Weight and Body Composition

Weight, fat mass and lean mass were measured all along the 10 weeks of dietary supplementation. Body weight increased in both control and hydrolysate fed mice over the 10 weeks of diet (time effect [*F*_(9, 126)_ = 25.93, *p* < 0.001]), in a diet-independent manner (diet effect [*F*_(1, 14)_ = 2.287, *p* = 0.153]) (data not shown). Fat mass gain and lean mass reduction were also similar between mice fed either with the control diet or the hydrolysate enriched-diet [*t*_(14)_ = 0.949, *p* = 0.359 and *t*_(14)_ = 0.688, *p* = 0.503, respectively] (data not shown).

### Short-Term and Long-Term Memory Evaluation

**Figure 3:**
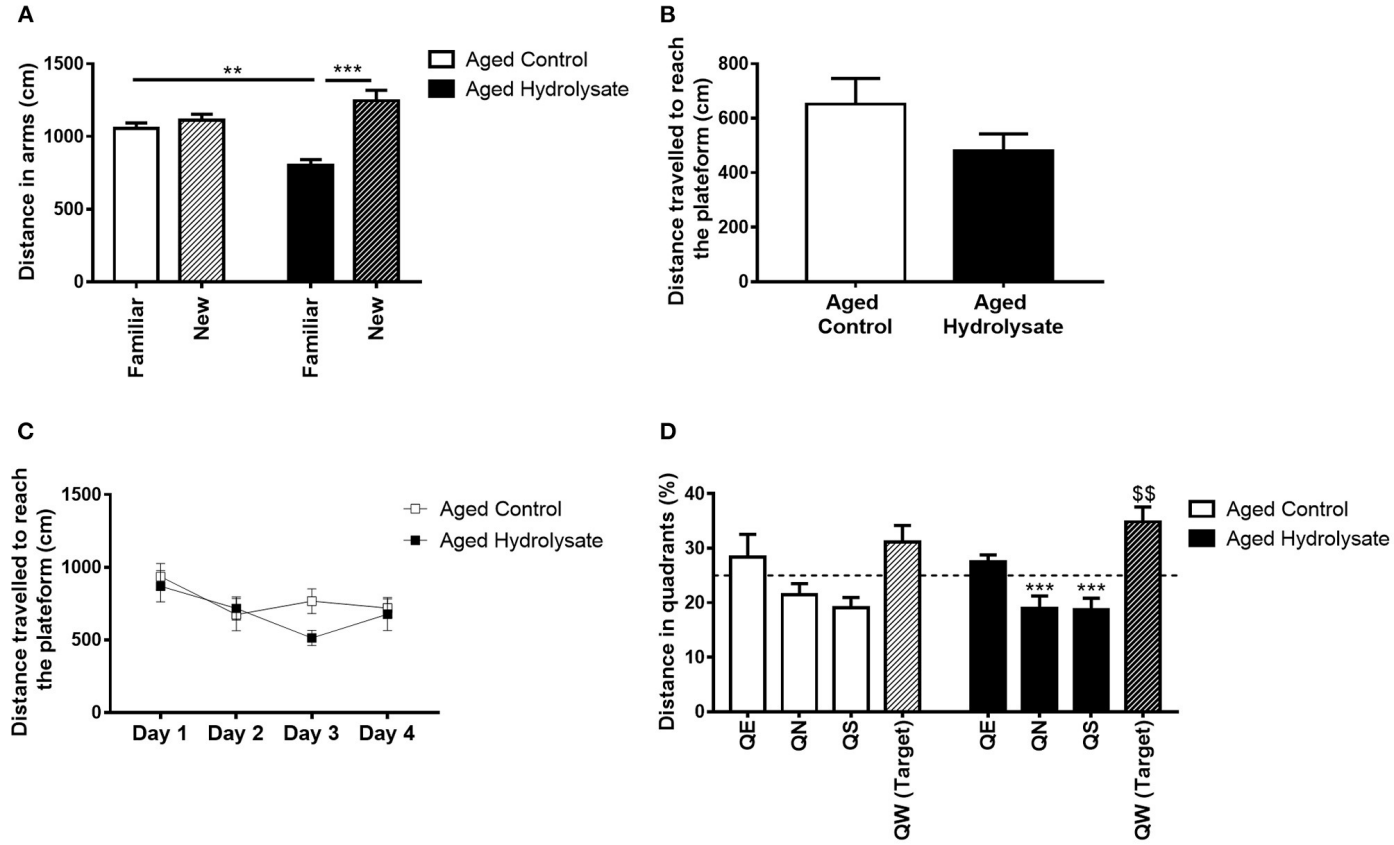
Effects of the fish hydrolysate supplementation on short-term memory, spatial learning and long-term memory. **(A)** Recognition of the new arm after a 1 h ITI in aged mice fed the control diet or the hydrolysate-enriched diet (^**^p < 0.01, ^***^p < 0.001 by 2-way ANOVA followed by Tukey *post-hoc* test). **(B)** Distance traveled during the cued learning **(C)** Distance covered to reach the platform over the 4 consecutive days of spatial learning (day effect *p* < 0.01 by 2-way ANOVA with repeated measures). **(D)** Percentage of distance traveled in quadrants during the probe test. The dotted line represents chance level (25%) [^$$^*p* < 0.01 vs. chance level by one-sample *t*-test. ^***^p < 0.001 compared to QW (Target) by One-way ANOVA and Dunnett's multiple comparison test; *n* = 8 per group]. Data are presented as mean ± SEM. QE, quadrant east; QN, quadrant north; QS, quadrant south; QW, quadrant west.

The effect of the hydrolysate supplementation on short-term spatial memory was assessed using a Y-maze test with a 1 h ITI. The 2-way ANOVA revealed an effect of arms [*F*_(1, 28)_ = 25.62, *p* < 0.001] but did not reveal any effect of the diet [*F*_(1, 28)_ = 1.451, *p* = 0.239]. However, the interaction between arms and diet was significant [*F*_(1, 28)_ = 15.08, *p* < 0.001]. The distance traveled in the familiar and in the new arm were not significantly different in control aged mice (Tukey *post-hoc* test: *p* = 0.838), characterizing short-term memory deficits. Furthermore, aged mice fed the hydrolysate diet traveled less distance in the familiar arm than control aged mice (Tukey *post-hoc* test: *p* < 0.01). These deficits were prevented in mice fed the hydrolysate diet, which traveled more distance in the new arm (Tukey *post-hoc* test: *p* < 0.001) (Figure 3A).

The effect of the hydrolysate supplementation on spatial learning and long-term memory was then assessed with the Morris water maze test. First, to evaluate their visuo-motor abilities, mice were trained to find a visible cued platform in the Morris water maze. Both groups traveled similar distances to reach the visible platform [*t*_(14)_ = 1.515, *p* = 0.152], meaning that they had similar visual abilities and did not display any impairment during the cued learning (Figure 3B). Mice were then submitted to the spatial learning. The 2-way ANOVA did not reveal any interaction [*F*_(3, 42)_ = 1.119, *p* = 0.352] or effect of the diet [*F*_(1, 14)_ = 1.098, *p* = 0.313]. However, both control and hydrolysate supplemented groups traveled significantly less distance over the 4 days of training [day effect, *F*_(3, 42)_ = 3.85, *p* < 0.05] indicating that learning was achieved (Figure 3C). Mice fed the hydrolysate enriched-diet did not show better performance than control mice (diet effect [*F*_(1, 14)_ = 1.098, *p* = 0.313]). Spatial memory was evaluated 72 h after the last day of spatial learning, during the probe test. A 2-way ANOVA was performed for the probe test and did not reveal any interaction [*F*_(3, 42)_ = 0.475, *p* = 0.702] and no effect of the diet [*F*_(1, 14)_ = 1.117, *p* = 0.308]. However, the analysis revealed a significant quadrant effect [*F*_(3, 42)_ = 10.47, *p* < 0.001]. One sample *t*-test compared to the chance level (25%) showed that control mice didn't travel more distance in the target quadrant [*t*_(7)_ = 1.533 *p* = 0.169], revealing that 72 h after the last day of training, aged control mice presented memory alterations (Figure 3D). The hydrolysate supplementation prevented this memory long-term memory deficit as shown in Figure 3D. Indeed, supplemented mice significantly traveled more distance in the target quadrant [*t*_(7)_ = 3.591, *p* < 0.01]. Furthermore, aged control mice failed to discriminate the target quadrant [*F*_(3, 28)_ = 2.164, *p* = 0.115] to the contrary of aged mice supplemented with the hydrolysate enriched-diet [*F*_(3, 28)_ = 12.61, *p* < 0.001]. They significantly differentiated QN and QS from the target quadrant (QN vs. QW: *p* < 0.001; QS vs. QW: *p* < 0.001) and tended to differentiate QE from the target quadrant (QE vs. QW: *p* = 0.061). The absence of differences between QE and QW could be explained by the freezing of the mice for the 10 first seconds of the probe test, suggesting the presence of anxiety-like behavior, which were corrected by the hydrolysate supplementation. The freezing of the mice can be due to the cold water but we used the temperature used by Morris (75). Freezing can also be a temporary stress related to immobility. This behavior is commonly observed, especially in aged animals, while the Morris water maze is test known to induce stress (83).

### Pro-inflammatory Cytokine Gene Expression in the Hippocampus

**Figure 4:**
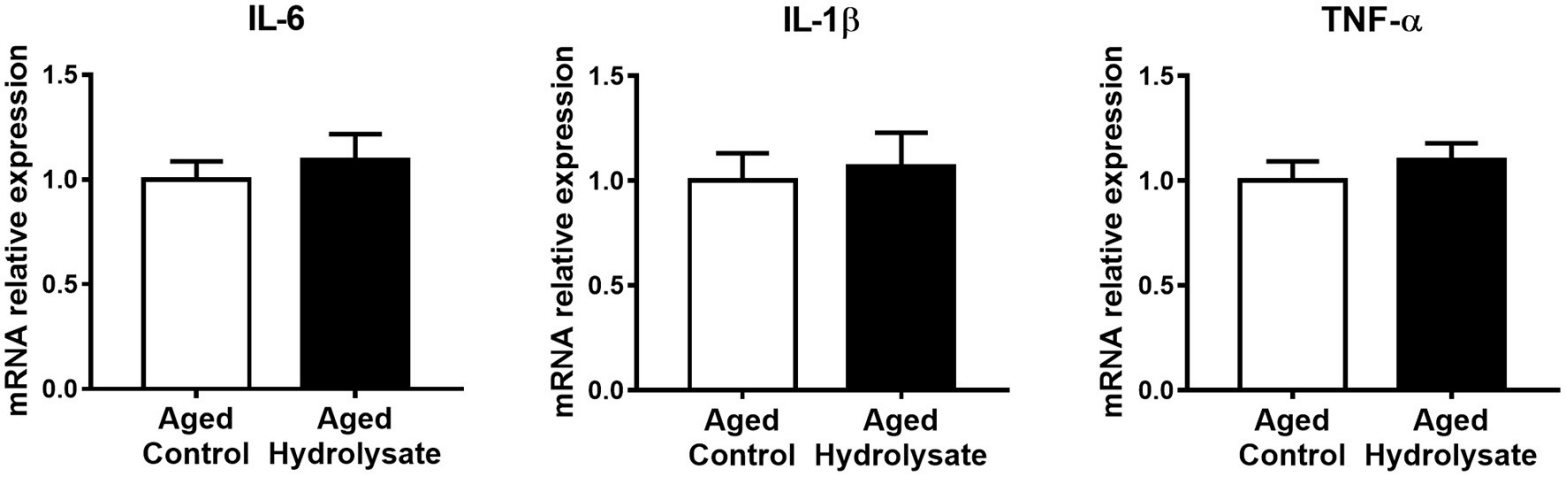
Effects of the fish hydrolysate supplementation on pro-inflammatory cytokine expression in the hippocampus. Pro-inflammatory cytokines IL-6, IL-1β, and TNF-α mRNA expression in the hippocampus of aged mice fed with the control diet or the hydrolysate-enriched diet for 10 weeks. *n* = 8 per group. Data are presented as mean ± SEM.

Gene expression of pro-inflammatory cytokines IL-6, IL-1β, and TNF-α was analyzed in the hippocampus of mice. The mRNA expression of IL-6, IL-1β, and TNF-α was not different between control and supplemented groups ([*t*_(14)_ = 0.601, *p* = 0.557]; [*t*_(14)_ = 0.326, *p* = 0.749], and [*t*_(14)_ = 0.821, *p* = 0.426], respectively) (Figure 4).

### Homeostatic and MGnD Microglial Signatures in the Hippocampus

**Figure 5:**
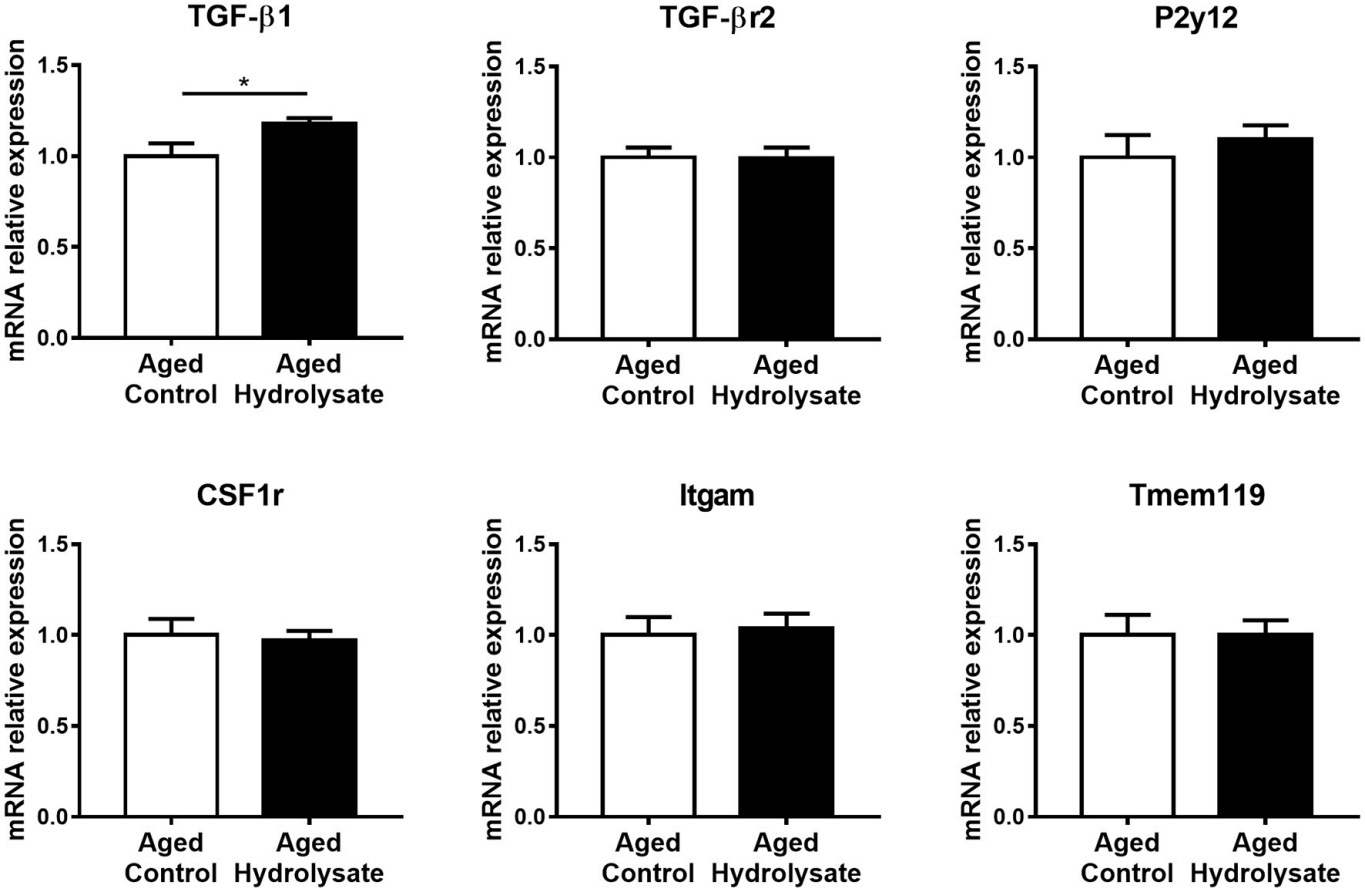
Effects of the fish hydrolysate supplementation on homeostatic microglial signature in the hippocampus. mRNA expression of homeostatic microglial markers TGFβ1, TGFβr2, P2y12, CSF1r, Itgam, and Tmem119 in the hippocampus of aged mice fed with the control diet or the hydrolysate-enriched diet for 10 weeks (^*^p < 0.05 by unpaired *t*-test; *n* = 8 per group). Data are presented as mean ± SEM.

The expression of genes that characterize the homeostatic microglial signature has been evaluated. Interestingly, the hydrolysate supplementation increased the expression of TGF-β1 compared to the control diet [*t*_(9.413)_ = 2.34, *p* < 0.05], which is essential for the maintenance of the homeostatic microglial signature (Figure 5). No differences were observed between mice fed with the control and the hydrolysate enriched-diet for TGF-βr2 [*t*_(14)_ = 0.07, *p* = 0.945], P2y12 [*t*_(14)_ = 0.7, *p* = 0.496], CSF1r [*t*_(14)_ = 0.279, *p* = 0.784], Itgam [*t*_(14)_ = 0.301, *p* = 0.768] and Tmem119 [*t*_(14)_ = 0.012, *p* = 0.991] (Figure 5).

**Figure 6:**
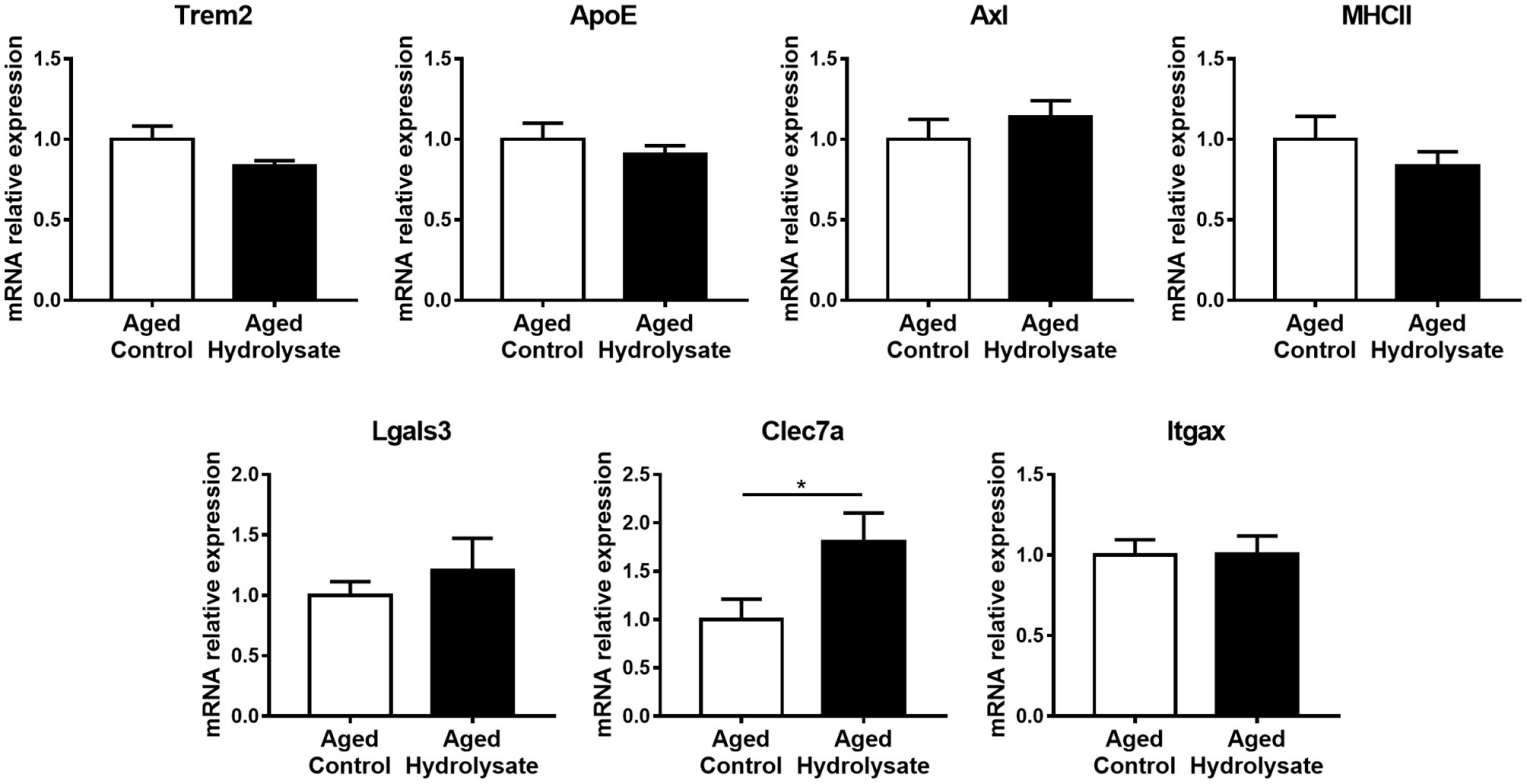
Effects of the fish hydrolysate supplementation on MGnD microglial signature in the hippocampus. mRNA expression of MGnD microglial signature Trem2, ApoE, Axl, MHCII, Lgals3, Clec7a, and Itgax in the hippocampus of aged mice fed with the control diet or the hydrolysate-enriched diet for 10 weeks (^*^p < 0.05 by unpaired *t*-test; *n* = 8 per group). Data are presented as mean ± SEM.

The expression of genes that characterize the MGnD microglial signature, occurring during aging, has also been evaluated in the same cerebral structure. Mice fed the hydrolysate enriched-diet displayed higher expression of Clec7a, which is involved in phagocytosis, compared to mice fed the control diet [*t*_(14)_ = 2.226, *p* < 0.05] (Figure 6). Moreover, the hydrolysate supplementation tended to decrease the expression of Trem2 [*t*_(14)_ = 1.84, *p* = 0.087], which is involved in the shift toward MGnD phenotype (Figure 6). No differences were observed between both control and hydrolysate supplemented groups for ApoE [*t*_(14)_ = 0.818, *p* = 0.427], Axl [*t*_(14)_ = 0.878, *p* = 0.395], MHCII [*t*_(14)_ = 0.987, *p* = 0.341], Lgals3 [*t*_(14)_ = 0.73, *p* = 0.478] and Itgax [*t*_(14)_ = 0.031, *p* = 0.976] (Figure 6).

### Hippocampal Clec7a-Positive Microglia

**Figure 7:**
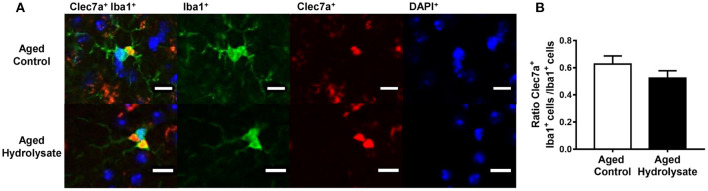
Effects of the fish hydrolysate supplementation on Clec7a^+^Iba1^+^/Iba1^+^ cells in the hippocampus. **(A)** Representative images of Clec7a^+^Iba1^+^/Iba1^+^ cells (double staining), Iba1^+^ cells (in green), Clec7a^+^ cells (in red) and DAPI^+^ cells (in blue) (scale bars: 10 μM) and **(B)** Number of positive cells for Clec7a and Iba1 staining in the hippocampus of aged mice fed with the control diet or the hydrolysate-enriched diet for 10 weeks (*n* = 8 per group). Data are presented as mean ± SEM.

We wanted to go further with the increased mRNA expression of Clec7a in the hippocampus of the aged hydrolysate group. We then performed immunohistochemical analysis on the number of cells positive for Clec7a within Iba1-positive microglia in the hippocampus (Figure 7A). The analysis revealed no significant difference between the number of Clec7a^+^ Iba1^+^ cells between mice fed either the control or the hydrolysate-enriched diet in the whole hippocampus [*t*_(8)_ = 1.265, *p* = 0.241] (Figure 7B).

### mRNA and Protein Expression of Intestinal Inflammation and Permeability Markers

**Figure 8:**
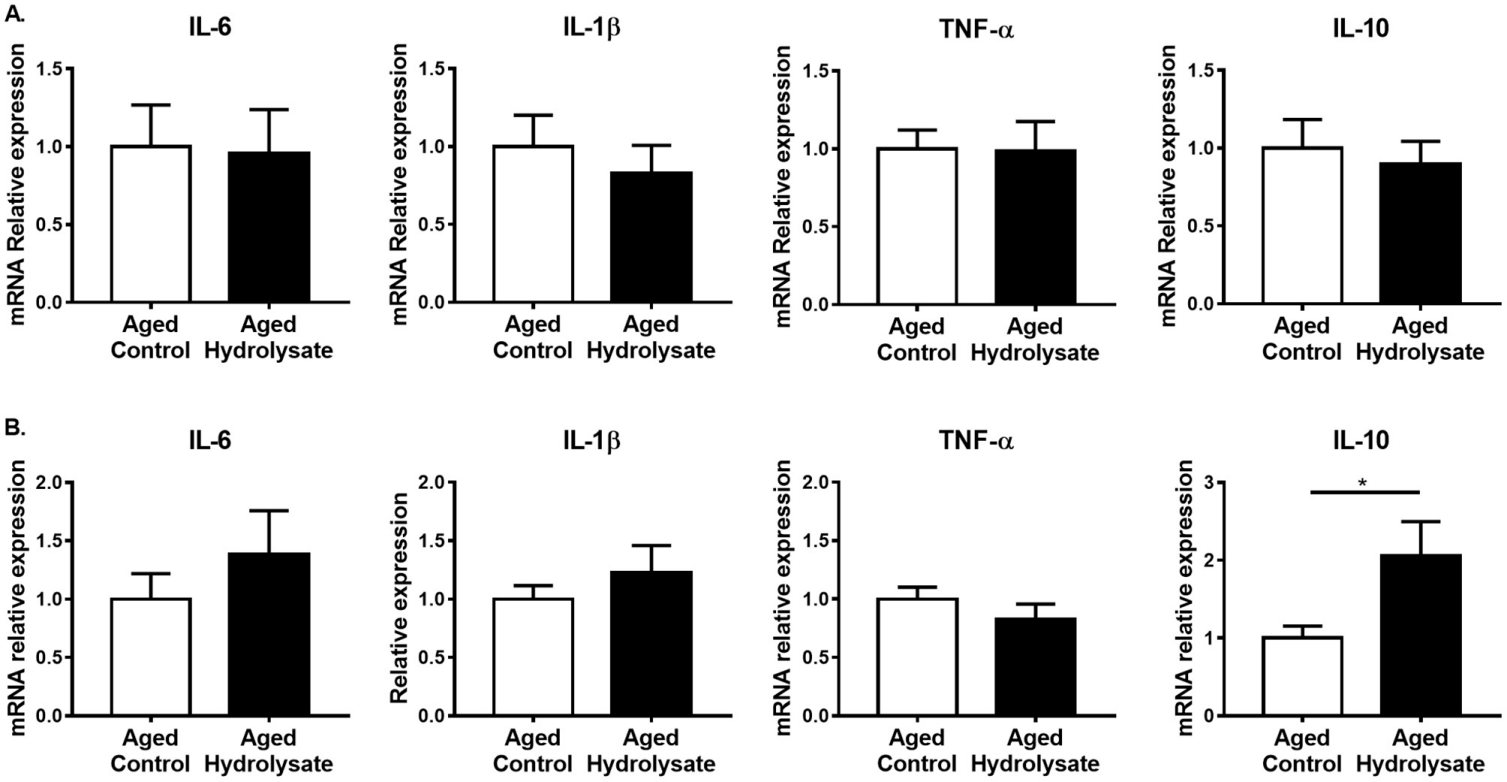
Effects of the fish hydrolysate supplementation on intestinal inflammation. Pro-inflammatory cytokines IL-6, IL-1β, and TNF-α and anti-inflammatory cytokine IL-10 mRNA expressions in **(A)** the ileum and **(B)** the colon of aged mice fed with the control diet or the hydrolysate-enriched diet for 10 weeks (^*^p < 0.05 by unpaired *t*-test; *n* = 8 per group). Data are presented as mean ± SEM.

Gut alterations related to aging lead to the production of inflammatory cytokines, thus contributing to chronic low-grade inflammation. Then, the effect of the hydrolysate supplementation was evaluated on inflammation in the ileum and the colon. In the ileum, gene expression of IL-6, IL-1β, TNF-α and IL-10 was not different between mice fed the control and the hydrolysate-enriched diet (IL-6 [*t*_(14)_ = 0.115, *p* = 0.911]; IL-1β [*t*_(14)_ = 0.637, *p* = 535]; TNF-α [*t*_(14)_ = 0.061, *p* = 0.952]; IL-10 [*t*_(14)_ = 0.436, *p* = 0.67]) (Figure 8A). In the colon, mRNA expression of IL-10 was significantly increased following the hydrolysate supplementation [*t*_(14)_ = 2.27, *p* < 0.05], suggesting an anti-inflammatory effect of the hydrolysate (Figure 8B). The mRNA expression of the other genes in the colon was comparable in the control and hydrolysate supplemented groups (IL-6 [*t*_(14)_ = 0.899, *p* = 0.384]; IL-1β [*t*_(14)_ = 0.832, *p* = 0.42]; TNF-α [*t*_(14)_ = 1.046, *p* = 0.313]) (Figure 8B).

**Figure 9:**
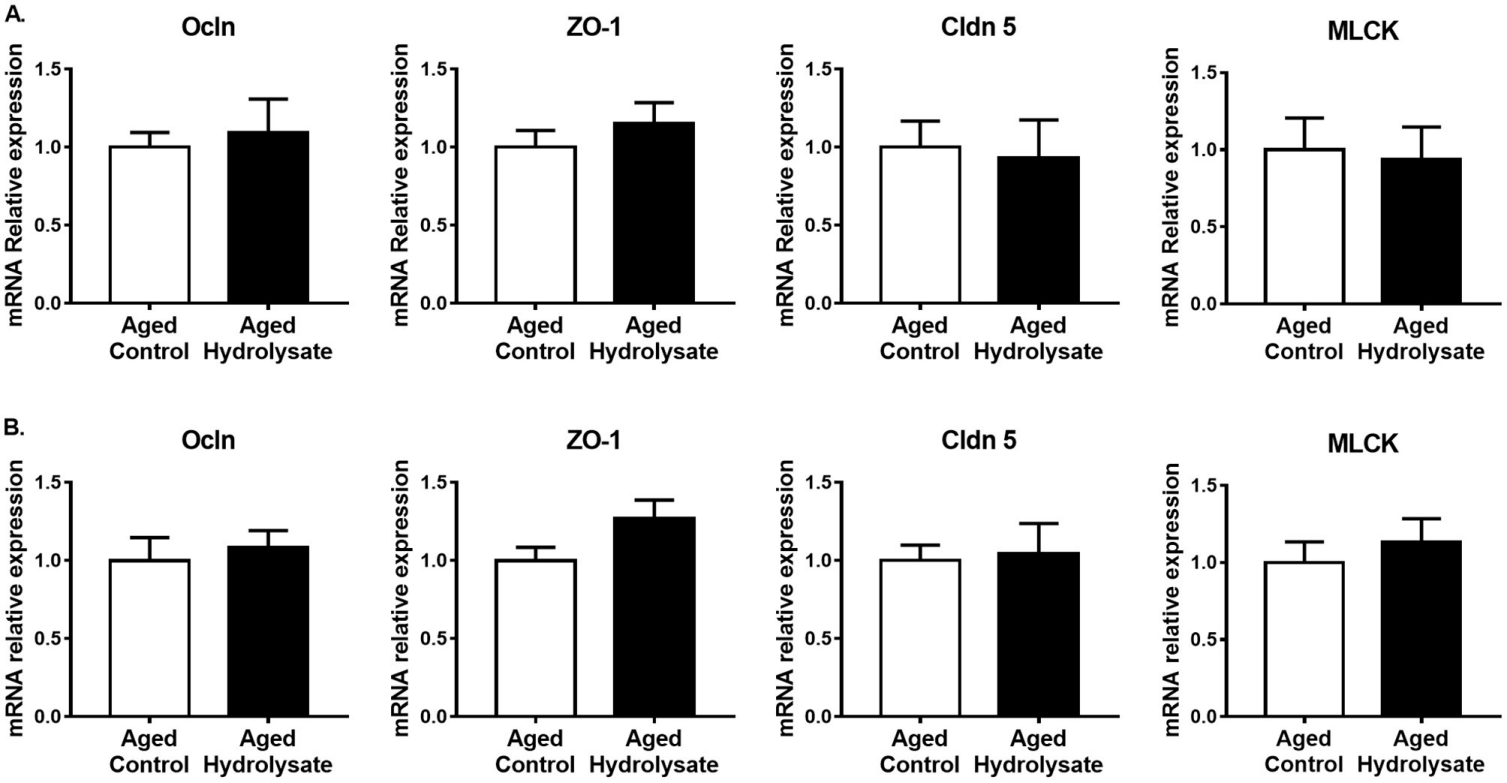
Effects of the fish hydrolysate supplementation on gene expression of intestinal permeability markers. Intestinal permeability markers Ocln, ZO-1, Cldn 5 and MLCK mRNA expressions in **(A)** the ileum and **(B)** the colon of aged mice fed with the control diet or the hydrolysate-enriched diet for 10 weeks. *n* = 8 per group. Data are presented as mean ± SEM.

Gut alterations related to aging, in addition to the production of inflammatory cytokines, lead to an increase of intestinal permeability. The effect of the hydrolysate supplementation was then evaluated on gene expression involved in ileum and colon permeability. In the ileum, gene expression of Ocln, ZO-1, Cldn5 and MLCK were not different between mice fed the control diet and mice fed the hydrolysate-enriched diet (Ocln [*t*_(14)_ = 0.398, *p* = 0.697]; ZO-1 [*t*_(14)_ = 0.87, *p* = 0.399]; Cldn5 [*t*_(14)_ = 0.24, *p* = 0.814]; MLCK [*t*_(14)_ = 0.212, *p* = 0.835]) (Figure 9A). In the colon, both control and supplemented groups displayed similar expression of Ocln [*t*_(14)_ = 0.448, *p* = 0.661], Cldn5 [*t*_(14)_ = 0.203, *p* = 0.843], and MLCK [*t*_(14)_ = 0.641, *p* = 0.532] (Figure 9B). The hydrolysate supplementation tended to increase the expression of ZO-1 [*t*_(14)_ = 1.891, *p* = 0.08] (Figure 9B).

**Figure 10:**
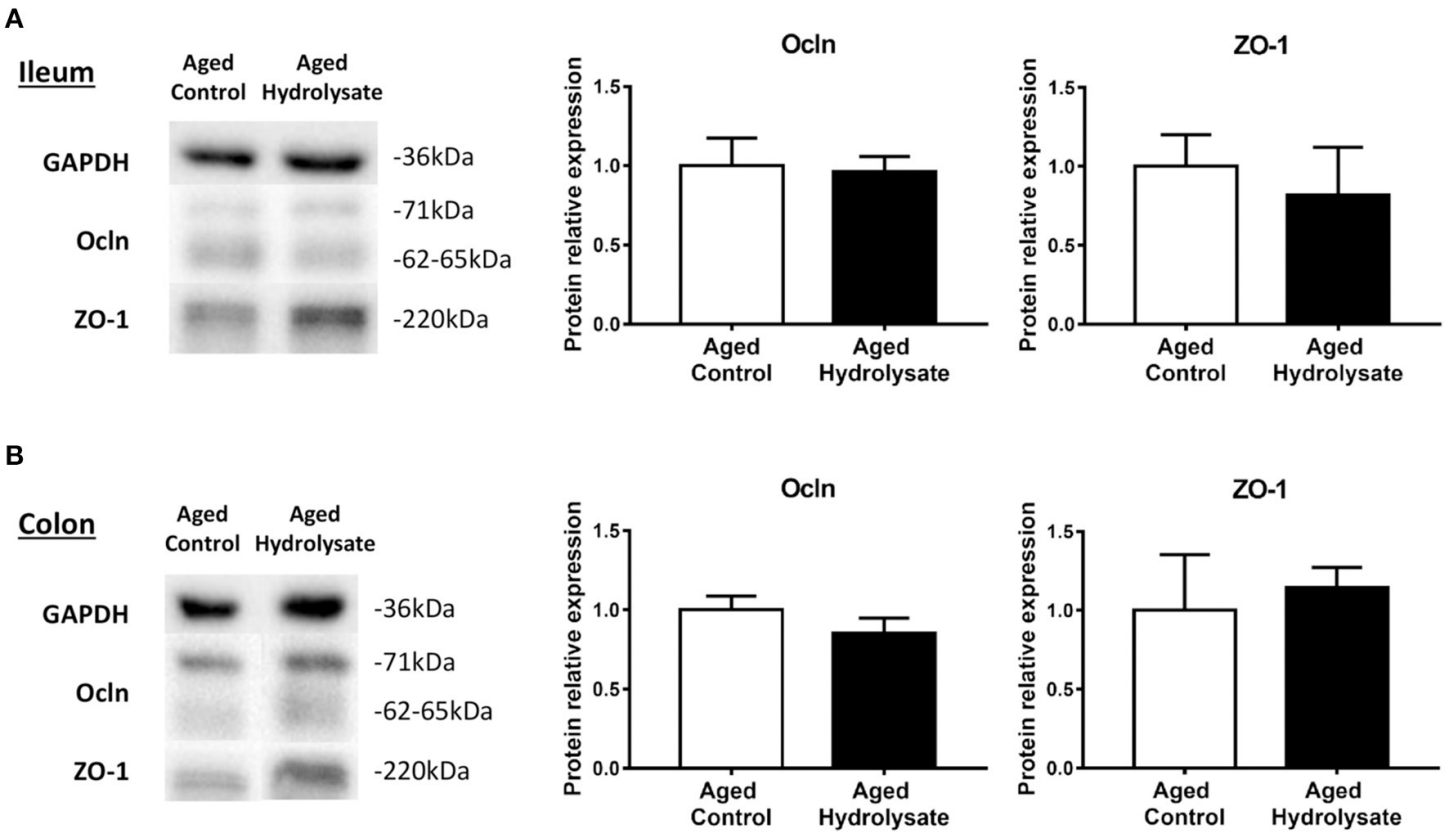
Effects of the fish hydrolysate supplementation on protein expression of intestinal permeability markers. Intestinal permeability markers ocln and ZO-1 protein expressions in **(A)** the ileum and **(B)** the colon of aged mice fed with the control diet or the hydrolysate-enriched diet for 10 weeks. *n* = 8 per group. Data are presented as mean ± SEM.

To go further, protein expression of Ocln and ZO-1 were assessed in the ileum and the colon. As shown in Figure 10 for Ocln, multiple reactive bands were observed at molecular weights of ~62–65 kDa for the lower molecular weight and 71 kDa for the higher molecular weight, representing the hyperphosphorylated form of the lower molecular weight form. Relative protein expression is represented as the ratio of protein expression to GAPDH. Total Ocln expression is represented as the ratio of the higher molecular weight form to the lower molecular weight form. No differences were observed between groups in the ileum (Ocln [*t*_(14)_ = 0.191; *p* = 0.851]; ZO-1 [*t*_(14)_ = 0.497; *p* = 0.627]) (Figure 10A) neither in the colon (Ocln [*t*_(14)_ = 1.122; *p* = 0.281]; ZO-1 [*t*_(8.81)_ = 0.384; *p* = 0.71]) (Figure 10B).

### Shift in Inflammatory, Intestinal Permeability, and Behavioral Marker Profile

**Figure 11:**
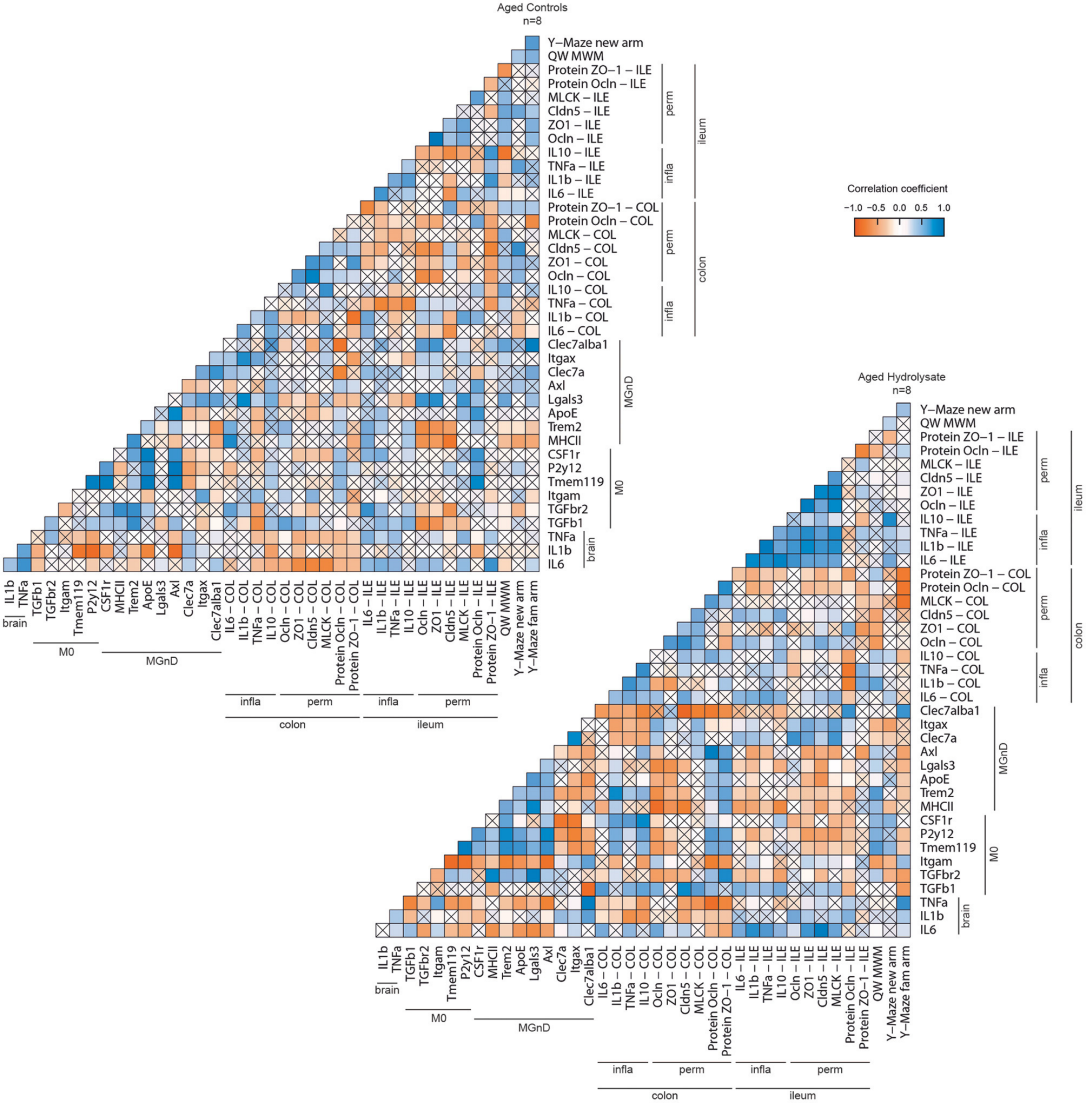
Correlation matrix of behavioral and biochemical parameters of each group. Positive correlations are represented in blue, negative correlations in red. No significant correlations are represented by a cross.

Correlation matrices were performed in aged control and aged hydrolysate groups. Overall, these correlation matrices revealed two different profiles based on the expression of hippocampal and intestinal inflammatory markers, intestinal permeability markers, behavioral assessment and immunological markers (Figure 11). Correlations between cognitive parameters and microglial markers were highlighted in both groups but to a lesser extent in the aged control group than in the aged hydrolysate group. In the aged hydrolysate group, the distance traveled in the new arm of the Y-Maze as well as in the target quadrant of the Morris water maze was positively correlated with genes involved in the homeostatic microglial signature, such as CSF1r, Tmem119, or P2y12, suggesting that the maintenance of this signature plays a role in cognitive function. In the aged control group, some markers of the MGnD microglial signature such as Trem2 or MHCII were negatively correlated with the distance traveled in the new arm of the Y-Maze. Likewise, the M0 marker P2y12 was positively correlated to the distance traveled in the target quadrant of the Morris water maze. Differences in the level of correlations between M0 and MGnD markers have been highlighted in the aged control group and the aged hydrolysate group. Indeed, in the aged hydrolysate group, the correlation matrix showed more significant positive than negative correlations, notably between the M0 markers TGF-βr2, Tmem119, P2y12, CSF1r and the MGnD markers MHCII, Trem2, ApoE, Lgals3, and Axl. In this group, negative correlations have also been highlighted between the M0 markers TGF-β1, Tmem119, P2y12, CSF1r and the MGnD markers Clec7a, Itgax, and Clec7a^+^Iba1^+^ cells. In comparison, the aged control group showed less significant correlations. Nevertheless, those correlations were similar to those observed in the aged hydrolysate group. The higher number of significant correlations in the aged hydrolysate group as compared to the aged control group suggested a higher microglial reactivity and switches between M0 and MGnD phenotype and a dysfunction in phenotype transition of microglial cells in the aged control group. Furthermore, in the aged hydrolysate group, markers of the MGnD microglial signature, such as Axl, Lgals3, ApoE, Trem2, and MHCII were negatively correlated with permeability markers, such as ZO-1, Cldn5, MLCK, Ocln (protein) in the ileum and Ocln, ZO-1, and Cldn5 in the colon, highlighting a link between microglial phenotypes and intestinal permeability. Moreover, different profiles were observed concerning correlations between intestinal inflammation and permeability. In the aged hydrolysate group, IL-10 in the ileum and the colon was positively correlated with ZO-1, Cldn5, and MLCK in the ileum and the colon whereas IL-10 in the ileum was negatively correlated to any variable in the aged control group. Concerning the pro-inflammatory cytokines, IL-6, IL-1β, and TNF-α in the ileum were positively correlated with Ocln, ZO-1, Cldn5, and MLCK in the ileum in the aged hydrolysate group. These correlations were not observed in the aged control group. We also noticed a positive correlation between colon inflammatory (IL-6, TNF-α) and permeability (Ocln, ZO-1, Cldn5) markers and brain IL-6. These results suggest a link between gut physiology and brain function.

## Discussion

Our results confirmed previous results obtained in our laboratory (73) and demonstrated that the hydrolysate supplementation prevents short- and long-term memory decline during aging. To better understand the mechanisms involved in the prevention of cognitive decline, we investigated the impact of the hydrolysate on microglial signature and on peripheral inflammation. We demonstrated that, without modulating pro-inflammatory cytokine expression, the fish hydrolysate supplementation modulated microglial signature. Indeed, mice supplemented with the fish hydrolysate displayed higher TGF-β1 expression, characteristic of the homeostatic microglial phenotype, higher expression of Clec7a, a marker of MGnD microglial signature involved in phagocytosis, and tended to express less Trem2. Our results represent a snapshot of the experimental conditions that we used during our study (i.e., population of microglia in the hippocampus of 17-months old mice supplemented or not). Although phenotypic changes can be transient and observed in a time-dependent manner, we can also suggest that these changes can be due to the fish hydrolysate supplementation that modulated microglia microenvironment. As shown by correlation matrices, aged mice supplemented with the hydrolysate enriched-diet displayed more significant and positive correlations between markers of the homeostatic microglial phenotype and markers of the MGnD phenotype, suggesting a higher reactivity of microglial cells as compared to the aged control group. The results are in accordance with a previous study that showed higher microglial reactivity in response to inflammation (84). Moreover, the hydrolysate supplementation promoted anti-inflammatory intestinal pathway and tended to prevent intestinal permeability alteration occurring during aging. The hydrolysate supplementation induced a shift in biochemical and behavioral marker profiles and appeared consequently as an interesting candidate to prevent cognitive decline during aging.

We highlighted a beneficial effect of the fish hydrolysate supplementation on TGF-β1 during aging, which is an anti-inflammatory cytokine largely involved in the regulation of inflammation, in cell proliferation, growth and differentiation as well as in neuroprotection (85). Moreover, protective effects against neuronal insults have also been observed before (10). We showed that TGF-β1 expression was higher in the hydrolysate supplementation group as compared to the control group. This effect could be linked to the enhancement of the cognitive performances in these mice. Indeed, defects in TGF-β1 have negative impact in physiological and pathological conditions. In normal aging in human, it was shown that a genetic variation within TGF-β1, leading to a lower production of TGF-β1, has a negative impact on functional and cognitive performance (85). In patients with Alzheimer's disease, impairment in TGF-β1 signaling is characterized by a reduction of TGF-β1 plasma levels and decreased receptor expression in neurons (85). In rodents, this cytokine seems to be involved in learning processes as demonstrated in mice and rats treated with a selective inhibitor of TGF-β1 signaling pathway (86, 87). These results suggest a possible role of TGF-β1 probably in the formation and remodeling of synapses (88).

In our study, aged mice fed the fish hydrolysate enriched-diet tended to express less Trem2 than aged control mice. This trend is interesting since this marker, which is highly expressed in glial cells, is involved in the switch from homeostatic microglial phenotype to MGnD phenotype (89, 90). Moreover, several studies have highlighted the beneficial effect of a Trem2 deficiency in aged mice on microglial activation, cognitive performance as well as hippocampal long-term potentiation, suggesting a potential detrimental role of Trem2 during physiological aging (89, 91). Further experimentations are needed to deepen the effect of the fish hydrolysate on Trem2 expression.

The expression of another gene associated to the MGnD phenotype, (Clec7a), was higher following the fish hydrolysate supplementation, although the number of positive microglia for Clec7a was not different from the aged control group. This could be the result of infiltrating cells, other than microglia, since this marker is expressed by macrophages as well as dendritic cells (92). It promotes microglial phagocytosis but can also be involved in pathogen recognition and the regulation of autophagy (93). In fine, it enhances the removal of cellular debris or damaged cell accumulation. Clec7a has been reported to enhance neuroinflammation when acting in synergy with Toll-like receptor 2 (94, 95) but in the regeneration of damaged CNS (96), *via* Syk-dependent signaling pathway. Syk is activated by phosphorylation into p-Syk, which, in turn activates signaling molecules such as NF-κB (95). It would be interesting to evaluate Syk and p-Syk expression to demonstrate the signaling pathways involved in the regulation of Clec7a by the hydrolysate.

Another possible mechanism of action of the fish hydrolysate during aging has been explored. We focused on the intestinal tract, which is involved in several physiological processes including nutrient intake as well as immune modulation (97). Indeed, a close link between gut inflammation, gut permeability and cognition has been demonstrated before (14). Age-related dysbiosis of the gut microbiota is known to be associated with aberrations of gut barrier integrity and enhanced pro-inflammatory cytokines. These changes impact the gut-brain axis thereby impairing neural, endocrine, and immunological signals between the gut and the brain *via* the enteric nervous system and could play a role in diseases of the CNS (98, 99). In aged rodents, several studies have reported an increased intestinal permeability to macromolecules and microbes, suggesting altered function and integrity of the intestinal barrier, leading to the leakage of microbial products in the circulation, thereby triggering systemic inflammation and contributing to cognitive impairments (12, 100–102). Recently, fecal microbiota transplant from aged mice to young mice has been shown to affect spatial learning and long-term memory, confirming the link between gut microbiota and cognitive function (103). It is also known that some nutrients can influence gut microbiota and functionality. In this study, aged mice fed the hydrolysate enriched-diet displayed higher colonic expression of IL-10 as compared to aged control mice. This is particularly interesting since IL-10 is a cytokine which plays a crucial role in the regulation of epithelial integrity as well as the regeneration of the colon (104, 105). In line with this, a positive correlation was also found between intestinal IL-10 and permeability markers ZO-1, Cldn5, and MLCK. Furthermore, IL-10 can interact with the intestinal microbiota to regulate epithelial function (106). The hydrolysate supplementation also tended to decrease intestinal permeability. The effects of n-3 LC-PUFAs on gut microbiota, intestinal permeability and immune function have recently been reviewed (66, 67, 70). EPA and DHA display significant beneficial effects on barrier integrity and intestinal inflammation as shown in *in vitro* and *in vivo* studies. Moreover, n-3 PUFAs can influence gut microbiota composition and, in turn, microbiota can impact the metabolism and absorption of n-3 PUFAs. However, less is known about the effects of low molecular weight peptides. Recently, the supplementation with small peptides from skipjack by-products have been shown to display anti-inflammatory effects in a mouse model of ulcerative colitis and to increase the diversity of the intestinal flora (107). These anti-inflammatory properties have also been observed in murine models of colitis supplemented with peptide derived from soy or oyster (63, 64). In addition, collagen peptides also protect the intestinal barrier function *in vitro, via* the regulation of ZO-1 and Ocln expression and distribution and the MLCK pathway (71, 72).

The effect of the fish hydrolysate on intestinal inflammation and permeability was highlighted in the colon but not in the ileum. This could be linked to differences in microbiota in these two intestinal tissues. Indeed, ileum and colon presented distinct microbiota suggesting different mechanisms of action (97, 108). Microbial signatures in colon and ileum are specific and may be differently modulated by the hydrolysate supplementation, as already shown in a study evaluating the effects of polyphenols on intestinal inflammation and gut microbiome signature (97). Moreover, aging induces change in microbiota diversity, which is linked to immune function and cognition (14). Comparisons between microbiota composition in the colon or the ileum would be interesting, as previous studies in humans have observed differences in composition and density between the microbiota of the distal ileum and the colon (109, 110). These microbiota compositions also changed with diet and we could speculate that it wasn't change similarly by the hydrolysate supplementation due to their basal composition.

This study has some limitations. A first limitation concerns the sample size, which could have been increased in order to increase the significance of the statistical tests. However, we had to comply with ethical regulations and our previous results have shown that the number of mice is sufficient to highlight beneficial effects of a hydrolysate supplementation on cognitive function as well as neuroinflammation (73, 84). We also acknowledge some potential bias for the multiple comparison analyses given the high risk of family-wise, thus given rise to potential false positives within the reported results. A second limitation concerns the lack of morphological analyses of microglia. Indeed, microglia morphology and function are closely related and morphological analyses would have given us information on their function. We chose to analyze the protein expression of Clec7a by immunofluorescence because it is involved in phagocytosis, which is enhanced during aging (10).

## Conclusion

This study provides further evidence for the understanding of the mechanisms of action of the marine hydrolysate containing n-3 LC-PUFAs and low molecular weight peptides on inflammation and cognitive functions during aging. The beneficial effects induced by the hydrolysate supplementation on behavioral and biochemical markers reinforce the innovative character of this hydrolysate on the prevention of age-related cognitive decline.

## Data Availability Statement

The original contributions presented in the study are included in the article/supplementary material, further inquiries can be directed to the corresponding author.

## Ethics Statement

The animal study was reviewed and approved by CE050 (Approval ID APAFIS#14144-2018041213072383).

## Author Contributions

VP, SL, AM, EB, A-LD, and CJ devised the project, the main conceptual ideas, and proof outline. MC, VP, SL, A-LD, and CJ conceived and designed experiments. MC and CL conducted research and analyzed data. MC and MDM performed statistical analysis. MC wrote the manuscript with support of A-LD and CJ. All authors have read and agreed to the published version of the manuscript.

## Funding

This work is part of the Brain Booster project which has been funded by Bpifrance and the Région Bretagne (DOS0049628/00).

## Conflict of Interest

MC, AM, and EB are employed by company Abyss Ingredients. The remaining authors declare that the research was conducted in the absence of any commercial or financial relationships that could be construed as a potential conflict of interest.

## Publisher's Note

All claims expressed in this article are solely those of the authors and do not necessarily represent those of their affiliated organizations, or those of the publisher, the editors and the reviewers. Any product that may be evaluated in this article, or claim that may be made by its manufacturer, is not guaranteed or endorsed by the publisher.

## References

[1] Cohen HJ Pieper CF Harris T Rao KM Currie MS. The association of plasma IL-6 levels with functional disability in community-dwelling elderly. J Gerontol A Biol Sci Med Sci. (1997) 52:M201–208. 10.1093/gerona/52A.4.M201 9224431

[2] Dik MG Jonker C Hack CE Smit JH Comijs HC Eikelenboom P. Serum inflammatory proteins and cognitive decline in older persons. Neurology. (2005) 64:1371–7. 10.1212/01.WNL.0000158281.08946.68 15851726

[3] Rafnsson SB Deary IJ Smith FB Whiteman MC Rumley A Lowe GDO. Cognitive decline and markers of inflammation and hemostasis: the Edinburgh Artery Study. J Am Geriatr Soc. (2007) 55:700–7. 10.1111/j.1532-5415.2007.01158.x 17493189

[4] Braida D Sacerdote P Panerai AE Bianchi M Aloisi AM Iosuè S. Cognitive function in young and adult IL (interleukin)-6 deficient mice. Behav Brain Res. (2004) 153:423–9. 10.1016/j.bbr.2003.12.018 15265638

[5] Buchanan JB Sparkman NL Chen J Johnson RW. Cognitive and neuroinflammatory consequences of mild repeated stress are exacerbated in aged mice. Psychoneuroendocrinology. (2008) 33:755–65. 10.1016/j.psyneuen.2008.02.013 18407425 PMC2580674

[6] Barrientos RM Frank MG Hein AM Higgins EA Watkins LR Rudy JW. Time course of hippocampal IL-1 beta and memory consolidation impairments in aging rats following peripheral infection. Brain Behav Immun. (2009) 23:46–54. 10.1016/j.bbi.2008.07.002 18664380 PMC2630971

[7] Krasemann S Madore C Cialic R Baufeld C Calcagno N El Fatimy R. The TREM2-APOE pathway drives the transcriptional phenotype of dysfunctional microglia in neurodegenerative diseases. Immunity. (2017) 47:566–81.e9. 10.1016/j.immuni.2017.08.008 28930663 PMC5719893

[8] Butovsky O Weiner HL. Microglial signatures and their role in health and disease. Nat Rev Neurosci. (2018) 19:622–35. 10.1038/s41583-018-0057-5 30206328 PMC7255106

[9] Hickman SE Kingery ND Ohsumi TK Borowsky ML Wang L Means TK. The microglial sensome revealed by direct RNA sequencing. Nat Neurosci. (2013) 16:1896–905. 10.1038/nn.3554 24162652 PMC3840123

[10] Butovsky O Jedrychowski MP Moore CS Cialic R Lanser AJ Gabriely G. Identification of a unique TGF-β-dependent molecular and functional signature in microglia. Nat Neurosci. (2014) 17:131–43. 10.1038/nn.3599 24316888 PMC4066672

[11] Holtman IR Raj DD Miller JA Schaafsma W Yin Z Brouwer N. Induction of a common microglia gene expression signature by aging and neurodegenerative conditions: a co-expression meta-analysis. Acta Neuropathol Commun. (2015) 3:31. 10.1186/s40478-015-0203-5 26001565 PMC4489356

[12] Thevaranjan N Puchta A Schulz C Naidoo A Szamosi JC Verschoor CP. Age-associated microbial dysbiosis promotes intestinal permeability, systemic inflammation, and macrophage dysfunction. Cell Host Microbe. (2017) 21:455–66.e4. 10.1016/j.chom.2017.03.002 28407483 PMC5392495

[13] Nagpal R Mainali R Ahmadi S Wang S Singh R Kavanagh K. Gut microbiome and aging: physiological and mechanistic insights. Nutr Healthy Aging. (2018) 4:267–85. 10.3233/NHA-170030 29951588 PMC6004897

[14] Cryan JF O'Riordan KJ Cowan CSM Sandhu KV Bastiaanssen TFS Boehme M. The microbiota-gut-brain axis. Physiol Rev. (2019) 99:1877–2013. 10.1152/physrev.00018.2018 31460832

[15] Madore C Yin Z Leibowitz J Butovsky O. Microglia, lifestyle stress, and neurodegeneration. Immunity. (2020) 52:222–40. 10.1016/j.immuni.2019.12.003 31924476 PMC7234821

[16] Gallagher M Rapp PR. The use of animal models to study the effects of aging on cognition. Annu Rev Psychol. (1997) 48:339–70. 10.1146/annurev.psych.48.1.339 9046563

[17] Deary IJ Corley J Gow AJ Harris SE Houlihan LM Marioni RE. Age-associated cognitive decline. Br Med Bull. (2009) 92:135–52. 10.1093/bmb/ldp033 19776035

[18] Streit WJ Braak H Xue Q-S Bechmann I. Dystrophic (senescent) rather than activated microglial cells are associated with tau pathology and likely precede neurodegeneration in Alzheimer's disease. Acta Neuropathol. (2009) 118:475–85. 10.1007/s00401-009-0556-6 19513731 PMC2737117

[19] von Bernhardi R Tichauer JE Eugenín J. Aging-dependent changes of microglial cells and their relevance for neurodegenerative disorders. J Neurochem. (2010) 112:1099–114. 10.1111/j.1471-4159.2009.06537.x 20002526

[20] Reichenberg A Yirmiya R Schuld A Kraus T Haack M Morag A. Cytokine-associated emotional and cognitive disturbances in humans. Arch Gen Psychiatry. (2001) 58:445–52. 10.1001/archpsyc.58.5.445 11343523

[21] Oitzl MS van Oers H Schöbitz B de Kloet ER. Interleukin-1 beta, but not interleukin-6, impairs spatial navigation learning. Brain Res. (1993) 613:160–3. 10.1016/0006-8993(93)90468-3 8348300

[22] Taepavarapruk P Song C. Reductions of acetylcholine release and nerve growth factor expression are correlated with memory impairment induced by interleukin-1 beta administrations: effects of omega-3 fatty acid EPA treatment. J Neurochem. (2010) 112:1054–64. 10.1111/j.1471-4159.2009.06524.x 19968753

[23] Aloe L Fiore M Probert L Turrini P Tirassa P. Overexpression of tumour necrosis factor alpha in the brain of transgenic mice differentially alters nerve growth factor levels and choline acetyltransferase activity. Cytokine. (1999) 11:45–54. 10.1006/cyto.1998.0397 10080878

[24] Golan H Levav T Mendelsohn A Huleihel M. Involvement of tumor necrosis factor alpha in hippocampal development and function. Cereb Cortex. (2004) 14:97–105. 10.1093/cercor/bhg108 14654461

[25] Patterson SL. Immune dysregulation and cognitive vulnerability in the aging brain: interactions of microglia, IL-1β, BDNF and synaptic plasticity. Neuropharmacology. (2015) 96:11–8. 10.1016/j.neuropharm.2014.12.020 25549562 PMC4475415

[26] Calder PC. Polyunsaturated fatty acids and inflammation. Biochem Soc Trans. (2005) 33:423–7. 10.1042/BST0330423 15787620

[27] Layé S Nadjar A Joffre C Bazinet RP. Anti-inflammatory effects of omega-3 fatty acids in the brain: physiological mechanisms and relevance to pharmacology. Pharmacol Rev. (2018) 70:12–38. 10.1124/pr.117.014092 29217656

[28] Joffre C Rey C Layé S. N-3 polyunsaturated fatty acids and the resolution of neuroinflammation. Front Pharmacol. (2019) 10:e01022. 10.3389/fphar.2019.01022 31607902 PMC6755339

[29] Kremer JM. n−3 Fatty acid supplements in rheumatoid arthritis. Am J Clin Nutr. (2000) 71:349s−51s. 10.1093/ajcn/71.1.349s 10617995

[30] Lamon-Fava S So J Mischoulon D Ziegler TR Dunlop BW Kinkead B. Dose- and time-dependent increase in circulating anti-inflammatory and pro-resolving lipid mediators following eicosapentaenoic acid supplementation in patients with major depressive disorder and chronic inflammation. Prostagland Leukotr Essen Fatty Acids. (2021) 164:102219. 10.1016/j.plefa.2020.102219 33316626 PMC7855824

[31] So J Wu D Lichtenstein AH Tai AK Matthan NR Maddipati KR. EPA and DHA differentially modulate monocyte inflammatory response in subjects with chronic inflammation in part *via* plasma specialized pro-resolving lipid mediators: a randomized, double-blind, crossover study. Atherosclerosis. (2021) 316:90–8. 10.1016/j.atherosclerosis.2020.11.018 33303222

[32] Kavanagh T Lonergan PE Lynch MA. Eicosapentaenoic acid and gamma-linolenic acid increase hippocampal concentrations of IL-4 and IL-10 and abrogate lipopolysaccharide-induced inhibition of long-term potentiation. Prostagland Leukotr Essen Fatty Acids. (2004) 70:391–7. 10.1016/j.plefa.2003.12.014 15041032

[33] Lonergan PE Martin DSD Horrobin DF Lynch MA. Neuroprotective actions of eicosapentaenoic acid on lipopolysaccharide-induced dysfunction in rat hippocampus. J Neurochem. (2004) 91:20–9. 10.1111/j.1471-4159.2004.02689.x 15379883

[34] Orr SK Palumbo S Bosetti F Mount HT Kang JX Greenwood CE. Unesterified docosahexaenoic acid is protective in neuroinflammation. J Neurochem. (2013) 127:378–93. 10.1111/jnc.12392 23919613 PMC4068707

[35] Dehkordi NG Noorbakhshnia M Ghaedi K Esmaeili A Dabaghi M. Omega-3 fatty acids prevent LPS-induced passive avoidance learning and memory and CaMKII-α gene expression impairments in hippocampus of rat. Pharmacol Rep. (2015) 67:370–5. 10.1016/j.pharep.2014.10.014 25712666

[36] Shi Z Ren H Huang Z Peng Y He B Yao X. Fish oil prevents lipopolysaccharide-induced depressive-like behavior by inhibiting neuroinflammation. Mol Neurobiol. (2017) 54:7327–34. 10.1007/s12035-016-0212-9 27815837

[37] Dong Y Xu M Kalueff AV Song C. Dietary eicosapentaenoic acid normalizes hippocampal omega-3 and 6 polyunsaturated fatty acid profile, attenuates glial activation and regulates BDNF function in a rodent model of neuroinflammation induced by central interleukin-1β administration. Eur J Nutr. (2018) 57:1781–91. 10.1007/s00394-017-1462-7 28523372

[38] Rey C Delpech JC Madore C Nadjar A Greenhalgh AD Amadieu C. Dietary n-3 long chain PUFA supplementation promotes a pro-resolving oxylipin profile in the brain. Brain Behav Immun. (2019) 76:17–27. 10.1016/j.bbi.2018.07.025 30086401

[39] Whalley LJ Fox HC Wahle KW Starr JM Deary IJ. Cognitive aging, childhood intelligence, and the use of food supplements: possible involvement of n-3 fatty acids. Am J Clin Nutr. (2004) 80:1650–7. 10.1093/ajcn/80.6.1650 15585782

[40] González S Huerta JM Fernández S Patterson AM Lasheras C. The relationship between dietary lipids and cognitive performance in an elderly population. Int J Food Sci Nutr. (2010) 61:217–25. 10.3109/09637480903348098 20001761

[41] Lee LK Shahar S Chin A-V Yusoff NAM. Docosahexaenoic acid-concentrated fish oil supplementation in subjects with mild cognitive impairment (MCI): a 12-month randomised, double-blind, placebo-controlled trial. Psychopharmacology (Berl). (2013) 225:605–12. 10.1007/s00213-012-2848-0 22932777

[42] Titova OE Sjögren P Brooks SJ Kullberg J Ax E Kilander L. Dietary intake of eicosapentaenoic and docosahexaenoic acids is linked to gray matter volume and cognitive function in elderly. Age (Dordr). (2013) 35:1495–505. 10.1007/s11357-012-9453-3 22791395 PMC3705118

[43] McNamara RK Kalt W Shidler MD McDonald J Summer SS Stein AL. Cognitive response to fish oil, blueberry, and combined supplementation in older adults with subjective cognitive impairment. Neurobiol Aging. (2018) 64:147–56. 10.1016/j.neurobiolaging.2017.12.003 29458842 PMC5822748

[44] Petursdottir AL Farr SA Morley JE Banks WA Skuladottir GV. Effect of dietary n-3 polyunsaturated fatty acids on brain lipid fatty acid composition, learning ability, and memory of senescence-accelerated mouse. J Gerontol A Biol Sci Med Sci. (2008) 63:1153–60. 10.1093/gerona/63.11.1153 19038829

[45] Kelly L Grehan B Chiesa AD O'Mara SM Downer E Sahyoun G. The polyunsaturated fatty acids, EPA and DPA exert a protective effect in the hippocampus of the aged rat. Neurobiol Aging. (2011) 32:2318.e1–15. 10.1016/j.neurobiolaging.2010.04.001 20570403

[46] Labrousse VF Nadjar A Joffre C Costes L Aubert A Grégoire S. Short-term long chain omega3 diet protects from neuroinflammatory processes and memory impairment in aged mice. PLoS ONE. (2012) 7:e36861. 10.1371/journal.pone.0036861 22662127 PMC3360741

[47] Cutuli D De Bartolo P Caporali P Laricchiuta D Foti F Ronci M. n-3 polyunsaturated fatty acids supplementation enhances hippocampal functionality in aged mice. Front Aging Neurosci. (2014) 6:e00220. 10.3389/fnagi.2014.00220 25202271 PMC4142709

[48] De Smedt-Peyrusse VD Sargueil F Moranis A Harizi H Mongrand S Layé S. Docosahexaenoic acid prevents lipopolysaccharide-induced cytokine production in microglial cells by inhibiting lipopolysaccharide receptor presentation but not its membrane subdomain localization. J Neurochem. (2008) 105:296–307. 10.1111/j.1471-4159.2007.05129.x 18021297

[49] Ajmone-Cat MA Salvatori ML Simone RD Mancini M Biagioni S Bernardo A. Docosahexaenoic acid modulates inflammatory and antineurogenic functions of activated microglial cells. J Neurosci Res. (2012) 90:575–87. 10.1002/jnr.22783 22057807

[50] Hjorth E Zhu M Toro VC Vedin I Palmblad J Cederholm T. Omega-3 fatty acids enhance phagocytosis of Alzheimer's disease-related amyloid-β 42 by human microglia and decrease inflammatory markers. J Alzheimer Dis. (2013) 35:697–713. 10.3233/JAD-130131 23481688

[51] Pettit LK Varsanyi C Tadros J Vassiliou E. Modulating the inflammatory properties of activated microglia with docosahexaenoic acid and aspirin. Lipids Health Dis. (2013) 12:16. 10.1186/1476-511X-12-16 23398903 PMC3663775

[52] Chen S Zhang H Pu H Wang G Li W Leak RK. n-3 PUFA supplementation benefits microglial responses to myelin pathology. Sci Rep. (2014) 4:7458. 10.1038/srep07458 25500548 PMC4264015

[53] Corsi L Dongmo BM Avallone R. Supplementation of omega 3 fatty acids improves oxidative stress in activated BV2 microglial cell line. Int J Food Sci Nutr. (2015) 66:293–9. 10.3109/09637486.2014.986073 25582176

[54] Inoue T Tanaka M Masuda S Ohue-Kitano R Yamakage H Muranaka K. Omega-3 polyunsaturated fatty acids suppress the inflammatory responses of lipopolysaccharide-stimulated mouse microglia by activating SIRT1 pathways. Biochim Biophys Acta Mol Cell Biol Lipids. (2017) 1862:552–60. 10.1016/j.bbalip.2017.02.010 28254441

[55] Aihara K Ishii H Yoshida M. Casein-derived tripeptide, Val-Pro-Pro (VPP), modulates monocyte adhesion to vascular endothelium. J Atheroscler Thromb. (2009) 16:594–603. 10.5551/jat.729 19907102

[56] Nakamura T Hirota T Mizushima K Ohki K Naito Y Yamamoto N. Milk-derived peptides, val-pro-pro and ile-pro-pro, attenuate atherosclerosis development in apolipoprotein E–deficient mice: a preliminary study. J Med Food. (2013) 16:396–403. 10.1089/jmf.2012.2541 23631494

[57] Zhang H Kovacs-Nolan J Kodera T Eto Y Mine Y. γ-Glutamyl cysteine and γ-glutamyl valine inhibit TNF-α signaling in intestinal epithelial cells and reduce inflammation in a mouse model of colitis *via* allosteric activation of the calcium-sensing receptor. Biochim Biophys Acta Mol Basis Dis. (2015) 1852:792–804. 10.1016/j.bbadis.2014.12.023 25558818

[58] Majumder K Mine Y Wu J. The potential of food protein-derived anti-inflammatory peptides against various chronic inflammatory diseases. J Sci Food Agric. (2016) 96:2303–11. 10.1002/jsfa.7600 26711001

[59] Lee SY Hur SJ. Mechanisms of neuroprotective effects of peptides derived from natural materials and their production and assessment. Compr Rev Food Sci Food Safety. (2019) 18:923–35. 10.1111/1541-4337.12451 33336993

[60] Min L-J Kobayashi Y Mogi M Tsukuda K Yamada A Yamauchi K. Administration of bovine casein-derived peptide prevents cognitive decline in Alzheimer disease model mice. PLoS ONE. (2017) 12:e0171515. 10.1371/journal.pone.0171515 28158298 PMC5291428

[61] Ahn C-B Cho Y-S Je J-Y. Purification and anti-inflammatory action of tripeptide from salmon pectoral fin byproduct protein hydrolysate. Food Chem. (2015) 168:151–6. 10.1016/j.foodchem.2014.05.112 25172694

[62] Montserrat de la Paz S Lemus-Conejo A Toscano R Pedroche J Millan F Millan-Linares MC. GPETAFLR, an octapeptide isolated from *Lupinus angustifolius* L. protein hydrolysate, promotes the skewing to the M2 phenotype in human primary monocytes. Food Funct. (2019) 10:3303–11. 10.1039/C9FO00115H 31094410

[63] Kovacs-Nolan J Zhang H Ibuki M Nakamori T Yoshiura K Turner PV. The PepT1-transportable soy tripeptide VPY reduces intestinal inflammation. Biochim Biophys Acta Gen Subjects. (2012) 1820:1753–63. 10.1016/j.bbagen.2012.07.007 22842481

[64] Hwang J-W Lee S-J Kim Y-S Kim E-K Ahn C-B Jeon Y-J. Purification and characterization of a novel peptide with inhibitory effects on colitis induced mice by dextran sulfate sodium from enzymatic hydrolysates of Crassostrea gigas. Fish Shellfish Immunol. (2012) 33:993–9. 10.1016/j.fsi.2012.08.017 22960100

[65] Calder PC. Marine omega-3 fatty acids and inflammatory processes: effects, mechanisms and clinical relevance. Biochim Biophys Acta. (2015) 1851:469–84. 10.1016/j.bbalip.2014.08.010 25149823

[66] Costantini L Molinari R Farinon B Merendino N. Impact of omega-3 fatty acids on the gut microbiota. Int J Mol Sci. (2017) 18:2645. 10.3390/ijms18122645 29215589 PMC5751248

[67] Fu Y Wang Y Gao H Li D Jiang R Ge L. Associations among dietary omega-3 polyunsaturated fatty acids, the gut microbiota, and intestinal immunity. Mediators Inflamm. (2021) 2021:e8879227. 10.1155/2021/8879227 33488295 PMC7801035

[68] Guinane CM Cotter PD. Role of the gut microbiota in health and chronic gastrointestinal disease: understanding a hidden metabolic organ. Therap Adv Gastroenterol. (2013) 6:295–308. 10.1177/1756283X13482996 23814609 PMC3667473

[69] Kaliannan K Wang B Li X-Y Kim K-J Kang JX. A host-microbiome interaction mediates the opposing effects of omega-6 and omega-3 fatty acids on metabolic endotoxemia. Sci Rep. (2015) 5:11276. 10.1038/srep11276 26062993 PMC4650612

[70] Durkin LA Childs CE Calder PC. Omega-3 polyunsaturated fatty acids and the intestinal epithelium—a review. Foods. (2021) 10:199. 10.3390/foods10010199 33478161 PMC7835870

[71] Chen Q Chen O Martins IM Hou H Zhao X Blumberg JB. Collagen peptides ameliorate intestinal epithelial barrier dysfunction in immunostimulatory Caco-2 cell monolayers *via* enhancing tight junctions. Food Funct. (2017) 8:1144–51. 10.1039/C6FO01347C 28174772

[72] Song W Chen Q Wang Y Han Y Zhang H Li B. Identification and structure–activity relationship of intestinal epithelial barrier function protective collagen peptides from alaska pollock skin. Mar Drugs. (2019) 17:450. 10.3390/md17080450 31370332 PMC6723256

[73] Chataigner M Mortessagne P Lucas C Pallet V Layé S Mehaignerie A. Dietary fish hydrolysate supplementation containing n-3 LC-PUFAs and peptides prevents short-term memory and stress response deficits in aged mice. Brain Behav Immun. (2021) 91:716–30. 10.1016/j.bbi.2020.09.022 32976934

[74] Dellu F Mayo W Cherkaoui J Le Moal M Simon H. A two-trial memory task with automated recording: study in young and aged rats. Brain Res. (1992) 588:132–9. 10.1016/0006-8993(92)91352-F 1393562

[75] Morris R. Developments of a water-maze procedure for studying spatial learning in the rat. J Neurosci Methods. (1984) 11:47–60. 10.1016/0165-0270(84)90007-4 6471907

[76] Mingam R Moranis A Bluthé R-M De Smedt-Peyrusse V Kelley KW Guesnet P. Uncoupling of interleukin-6 from its signalling pathway by dietary n-3-polyunsaturated fatty acid deprivation alters sickness behaviour in mice. Eur J Neurosci. (2008) 28:1877–86. 10.1111/j.1460-9568.2008.06470.x 18973601 PMC2769572

[77] Rey C Nadjar A Buaud B Vaysse C Aubert A Pallet V. Resolvin D1 and E1 promote resolution of inflammation in microglial cells *in vitro*. Brain Behav Immun. (2016) 55:249–59. 10.1016/j.bbi.2015.12.013 26718448

[78] Simões AE Pereira DM Amaral JD Nunes AF Gomes SE Rodrigues PM. Efficient recovery of proteins from multiple source samples after trizol^®^ or trizol^®^LS RNA extraction and long-term storage. BMC Genomics. (2013) 14:181. 10.1186/1471-2164-14-181 23496794 PMC3620933

[79] R Core Team. R: A Language and Environment for Statistical Computing. R Foundation for Statistical Computing, Vienna (2013). Available online at: http://www.R-project.org/ (accessed November 10, 2021).

[80] Murtagh F Legendre P. Ward's hierarchical agglomerative clustering method: which algorithms implement ward's criterion? J Classif. (2014) 31:274–95. 10.1007/s00357-014-9161-z

[81] Ward JH. Hierarchical grouping to optimize an objective function. J Am Stat Assoc. (1963) 58:236–44. 10.1080/01621459.1963.10500845

[82] Greenhouse SW Geisser S. On methods in the analysis of profile data. Psychometrika. (1959) 24:95–112. 10.1007/BF02289823

[83] Harrison FE Hosseini AH McDonald MP. Endogenous anxiety and stress responses in water maze and Barnes maze spatial memory tasks. Behav Brain Res. (2009) 198:247–51. 10.1016/j.bbr.2008.10.015 18996418 PMC2663577

[84] Chataigner M Martin M Lucas C Pallet V Layé S Mehaignerie A. Fish hydrolysate supplementation containing n-3 long chain polyunsaturated fatty acids and peptides prevents lps-induced neuroinflammation. Nutrients. (2021) 13:824. 10.3390/nu13030824 33801489 PMC7998148

[85] Fraga VG Guimarães HC Lara VP Teixeira AL Barbosa MT Carvalho MG. TGF-β1 codon 10 T>C polymorphism influences short-term functional and cognitive decline in healthy oldest-old individuals: the pietà study. J Alzheimers Dis. (2015) 48:1077–81. 10.3233/JAD-150397 26402106

[86] Caraci F Gulisano W Guida CA Impellizzeri AAR Drago F Puzzo D. A key role for TGF-β1 in hippocampal synaptic plasticity and memory. Sci Rep. (2015) 5:11252. 10.1038/srep11252 26059637 PMC4462026

[87] Arkhipov VI Pershina EV Levin SG. Deficiency of transforming growth factor-β signaling disrupts memory processes in rats. Neuroreport. (2018) 29:353–5. 10.1097/WNR.0000000000000971 29334566

[88] Diniz LP Matias I Siqueira M Stipursky J Gomes FCA. Astrocytes and the TGF-β1 pathway in the healthy and diseased brain: a double-edged sword. Mol Neurobiol. (2019) 56:4653–79. 10.1007/s12035-018-1396-y 30377983

[89] Qu W Li L. Loss of TREM2 confers resilience to synaptic and cognitive impairment in aged mice. J Neurosci. (2020) 40:9552–63. 10.1523/JNEUROSCI.2193-20.2020 33139402 PMC7726534

[90] Wei M-D Wang Y-H Lu K Lv B-J Wang Y Chen W-Y. Ketamine reverses the impaired fear memory extinction and accompanied depressive-like behaviors in adolescent mice. Behav Brain Res. (2020) 379:112342. 10.1016/j.bbr.2019.112342 31705920

[91] Linnartz-Gerlach B Bodea L-G Klaus C Ginolhac A Halder R Sinkkonen L. TREM2 triggers microglial density and age-related neuronal loss. Glia. (2019) 67:539–50. 10.1002/glia.23563 30548312 PMC6590266

[92] Brown GD Gordon S. A new receptor for β-glucans. Nature. (2001) 413:36–7. 10.1038/35092620 11544516

[93] Brown GD. Dectin-1: a signalling non-TLR pattern-recognition receptor. Nat Rev Immunol. (2006) 6:33–43. 10.1038/nri1745 16341139

[94] Rogers NC Slack EC Edwards AD Nolte MA Schulz O Schweighoffer E. Syk-dependent cytokine induction by dectin-1 reveals a novel pattern recognition pathway for C type lectins. Immunity. (2005) 22:507–17. 10.1016/j.immuni.2005.03.004 15845454

[95] Ye X-C Hao Q Ma W-J Zhao Q-C Wang W-W Yin H-H. Dectin-1/Syk signaling triggers neuroinflammation after ischemic stroke in mice. J Neuroinflammation. (2020) 17:17. 10.1186/s12974-019-1693-z 31926564 PMC6954534

[96] Baldwin KT Carbajal KS Segal BM Giger RJ. Neuroinflammation triggered by β-glucan/dectin-1 signaling enables CNS axon regeneration. Proc Natl Acad Sci USA. (2015) 112:2581–6. 10.1073/pnas.1423221112 25675510 PMC4345569

[97] Saccon TD Nagpal R Yadav H Cavalcante MB Nunes AD de C. Senolytic combination of dasatinib and quercetin alleviates intestinal senescence and inflammation and modulates the gut microbiome in aged mice. J Gerontol A. (2021) 76:1895–905. 10.1093/gerona/glab002 33406219 PMC8514064

[98] Collins SM Surette M Bercik P. The interplay between the intestinal microbiota and the brain. Nat Rev Microbiol. (2012) 10:735–42. 10.1038/nrmicro2876 23000955

[99] Luna RA Foster JA. Gut brain axis: diet microbiota interactions and implications for modulation of anxiety and depression. Curr Opin Biotechnol. (2015) 32:35–41. 10.1016/j.copbio.2014.10.007 25448230

[100] Mullin JM Valenzano MC Verrecchio JJ Kothari R. Age- and diet-related increase in transepithelial colon permeability of fischer 344 rats. Dig Dis Sci. (2002) 47:2262–70. 10.1023/A:1020191412285 12395899

[101] Annaert P Brouwers J Bijnens A Lammert F Tack J Augustijns P. *Ex vivo* permeability experiments in excised rat intestinal tissue and *in vitro* solubility measurements in aspirated human intestinal fluids support age-dependent oral drug absorption. Eur J Pharm Sci. (2010) 39:15–22. 10.1016/j.ejps.2009.10.005 19837159

[102] Cattaneo A Cattane N Galluzzi S Provasi S Lopizzo N Festari C. Association of brain amyloidosis with pro-inflammatory gut bacterial taxa and peripheral inflammation markers in cognitively impaired elderly. Neurobiol Aging. (2017) 49:60–8. 10.1016/j.neurobiolaging.2016.08.019 27776263

[103] D'Amato A Di Cesare Mannelli L Lucarini E Man AL Le Gall G Branca JJV. Faecal microbiota transplant from aged donor mice affects spatial learning and memory *via* modulating hippocampal synaptic plasticity- and neurotransmission-related proteins in young recipients. Microbiome. (2020) 8:140. 10.1186/s40168-020-00914-w 33004079 PMC7532115

[104] Pull SL Doherty JM Mills JC Gordon JI Stappenbeck TS. Activated macrophages are an adaptive element of the colonic epithelial progenitor niche necessary for regenerative responses to injury. Proc Natl Acad Sci USA. (2005) 102:99–104. 10.1073/pnas.0405979102 15615857 PMC544052

[105] Quiros M Nishio H Neumann PA Siuda D Brazil JC Azcutia V. Macrophage-derived IL-10 mediates mucosal repair by epithelial WISP-1 signaling. J Clin Invest. (2017) 127:3510–20. 10.1172/JCI90229 28783045 PMC5669557

[106] Zheng L Kelly CJ Battista KD Schaefer R Lanis JM Alexeev EE. Microbial-derived butyrate promotes epithelial barrier function through il-10 receptor–dependent repression of claudin-2. J Immunol. (2017) 199:2976–84. 10.4049/jimmunol.1700105 28893958 PMC5636678

[107] Wang Z Ying X Gao P Wang C Wang Y Yu X. Anti-inflammatory activity of a peptide from skipjack (*Katsuwonus pelamis*). Mar Drugs. (2019) 17:582. 10.3390/md17100582 31614893 PMC6835902

[108] Naftali T Reshef L Kovacs A Porat R Amir I Konikoff FM. Distinct microbiotas are associated with ileum-restricted and colon-involving Crohn's disease. Inflamm Bowel Dis. (2016) 22:293–302. 10.1097/MIB.0000000000000662 26752462

[109] Dieterich W Schink M Zopf Y. Microbiota in the gastrointestinal tract. Med Sci (Basel). (2018) 6:116. 10.3390/medsci6040116 30558253 PMC6313343

[110] Villmones HC Haug ES Ulvestad E Grude N Stenstad T Halland A. Species level description of the human ileal bacterial microbiota. Sci Rep. (2018) 8:4736. 10.1038/s41598-018-23198-5 29549283 PMC5856834

